# F-actin architecture determines constraints on myosin thick filament motion

**DOI:** 10.1038/s41467-022-34715-6

**Published:** 2022-11-16

**Authors:** Camelia G. Muresan, Zachary Gao Sun, Vikrant Yadav, A. Pasha Tabatabai, Laura Lanier, June Hyung Kim, Taeyoon Kim, Michael P. Murrell

**Affiliations:** 1 Department of Biomedical Engineering, Yale University, 55 Prospect Street, New Haven, CT 06511 USA; 2 Systems Biology Institute, Yale University, 850 West Campus Drive, West Haven, CT 06516 USA; 3 Department of Physics, Yale University, 217 Prospect Street, New Haven, CT 06511 USA; 4 Weldon School of Biomedical Engineering, Purdue University, 206S. Martin Jischke Drive, West Lafayette, IN 47907 USA

**Keywords:** Deformation dynamics, Biological physics, Cytoskeletal proteins

## Abstract

Active stresses are generated and transmitted throughout diverse F-actin architectures within the cell cytoskeleton, and drive essential behaviors of the cell, from cell division to migration. However, while the impact of F-actin architecture on the transmission of stress is well studied, the role of architecture on the ab initio generation of stresses remains less understood. Here, we assemble F-actin networks in vitro, whose architectures are varied from branched to bundled through F-actin nucleation via Arp2/3 and the formin mDia1. Within these architectures, we track the motions of embedded myosin thick filaments and connect them to the extent of F-actin network deformation. While mDia1-nucleated networks facilitate the accumulation of stress and drive contractility through enhanced actomyosin sliding, branched networks prevent stress accumulation through the inhibited processivity of thick filaments. The reduction in processivity is due to a decrease in translational and rotational motions constrained by the local density and geometry of F-actin.

## Abstract (web summary)

While the impact of F-actin architecture on stress transmission is well studied, the role of architecture on stress generation remains unclear. Here authors use in vitro model and show that distinct organizations constrain myosin motion.

## Introduction

Diverse actomyosin architectures coordinate contractility and force generation to drive changes in cell shape and internal cytoskeletal flows^1–4^. The architecture of the F-actin cytoskeleton is largely determined by the activity of the Arp2/3 complex and the formin mDia1^5,6^. The Arp2/3 complex branches short actin filaments, generating dendritic structures that assemble into large-scale networks^7,8^. By contrast, formin elongates linear structures^9^ which bundle and crosslink into networks^10,11^. In combination, they nucleate a composite F-actin material with variable dynamical and mechanical properties^1,3,6,12,13^.

In vivo, mDia1 and Arp2/3 contribute to the assembly of the mammalian cell cortex, a dense submembraneous F-actin network^14–16^. In non-adherent cells, the organization of the cortex is structurally isotropic on micrometer-lengthscale, as the pore size is small (~50 nm)^1^. Inhibition of Arp2/3 results in structural anisotropies^17^, large pores (~300 nm) and spatial variation in actin density^5^. Further, the cortex generates mechanical tension through the activity of myosin II^18,19^. The presence of Arp2/3-branched actin is concomitant with a reduction of cortical tension, as its depletion results in an increase in mechanical stress^20,21^. In adherent cells, Arp2/3 and formin structures spatially segregate into branched F-actin in the lamellipodium and myosin-rich bundled arrays in the lamella^22,23^. At the leading edge, Arp2/3 assembly generates protrusive forces^24,25^, while at the lamellipodium-lamellar border, Arp2/3 branching opposes actin retrograde flow^26,27^. Further, elevated Arp2/3 activity biases the cell towards lamellipodial protrusion versus contractile blebbing as motile mechanisms^28^. Thus, in both adherent and non-adherent cells, the presence of Arp2/3 is often associated with decreased myosin-based F-actin contractility^12^. However, it is unclear whether branched F-actin inhibits contractility through its geometric or mechanical characteristics directly. For example, Arp 2/3 depletion may induce de novo assembly of formin-nucleated actin or induce other downstream effects^5,13,29^. Myosin may then preferentially associate with formin-based structures, and thus contractility is enhanced with Arp2/3 depletion as myosin is recruited to bundles^30^. Therefore, biochemical, and genetic regulation obscure the geometric or mechanical impacts of F-actin architecture on the generation of myosin-generated active stresses.

In vitro, the reconstitution of actomyosin contractility has been parameterized by a balance between myosin-generated mechanical stress and F-actin network connectivity in networks assembled from purified protein^31–37^. Branched F-actin forms a gel^1,38^ and may facilitate contractility by providing efficient percolation compared to uncrosslinked networks^25,39,40^. However, F-actin is debranched under mechanical force^41^, indicating that network percolation is load-dependent^42^. Furthermore, local branched F-actin is not anti-parallel, and may be antagonistic to actomyosin sliding^43–45^. However, there is little information on how the local geometric and mechanical environment influences the motions of myosin thick filaments.

Here, we assemble thin ‘cortex-like’ actin networks in vitro, by nucleating actin from either Arp2/3 or the formin mDia1 with variable coupling to a phospholipid bilayer. In varying the nucleator type and concentration while maintaining a constant actin concentration, we alter the architecture of the resultant network, including its geometric and mechanical properties. Then, we decorate the networks with thick filaments of myosin II, and use high resolution multi-particle tracking (MPT) to quantify their motions. We then correlate these motions with the extent of local deformation and large-scale contraction of the F-actin network. In connecting these local myosin motions to large-scale F-actin network behaviors, we identify how diverse F-actin architectures coordinate the mechanical and dynamical stability of the actomyosin cytoskeleton.

## Results

### F-actin nucleation determines disparate and diverse steady-state network architectures

**Fig. 1:**
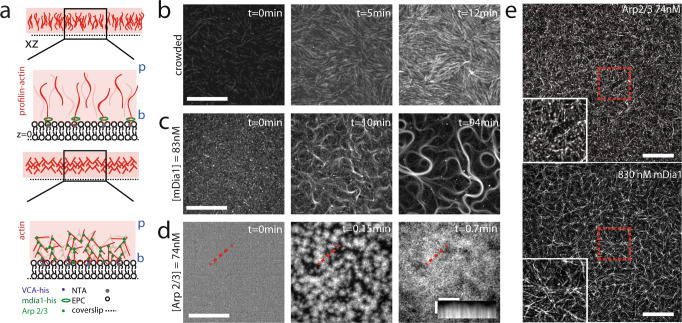
Reconstitution of F-actin networks with variable architectures. **a** Diagram of F-actin nucleated and grown from a phospholipid bilayer with either mDia1 (green ellipses) coupled to nickel lipid (NTA) (top) or Arp2/3 (green dots) activated by VCA (blue dots) coupled to nickel lipid (bottom). (‘b’ is barbed end, and ‘p’ is pointed end of F-actin. **b** Pre-polymerized F-actin crowded to the surface of the bilayer over time. Scalebar is 20 μm. **c** 83 nM mDia1-nucleated F-actin growth over time. Scalebar is 20 μm. **d** 74 nM Arp2/3-nucleated F-actin growth over time. Scalebar is 20 μm. Scalebar for inset kymograph is 5 μm (horizontal), and 30 s (vertical). **e** Super-resolution image of 74 nM Arp2/3-nucleated (top) and 830 nM mDia1-nucleated (bottom) F-actin networks. Experiments were repeated 3 times independently with similar results. Scale bar is 5 μm.

F-actin networks are nucleated from a phospholipid bilayer adjacent to a coverslip (Fig. 1a, Methods). The F-actin nucleators, along with their concentration at the bilayer surface are varied, while maintaining a constant overall actin concentration. To assemble F-actin networks nucleated by the formin mDia1, Histidine-tagged (HST) mDia1 is bound to nickel lipid (Ni) embedded within an Egg PC bilayer. 83 nM or 830 nM mDia1 are added to the sample chamber to bind with nickel lipid. Subsequently, 0.7 μM G-actin and 2.1 µM profilin are added to induce polymerization. To assemble F-actin networks nucleated by Arp 2/3, a WASp fragment, VCA, is also Histidine-tagged and bound to the bilayer. In this case, 0.74 nM, 7.4 nM, or 74 nM Arp2/3 is then bound and activated by VCA, also at 0.7 μM concentration of G-actin (15% Rhodamin labeled). In both cases, 0.25% methylcellulose is added to aid in polymerization and maintain a thin 3-D network^46,47^.

F-actin nucleation by mDia1 results in the growth of linear actin filaments from the surface of the bilayer. The filaments entangle into a thin network whose density and structure depend upon concentration of mDia1 at the surface (Fig. 1a and Supplementary Fig. 3). At 83 nM mDia1, actin filaments lengthen and align into dense bundles with high curvatures (Fig. 1c, Supplementary Movie 1). The network structure is irregular as the bundles vary in thickness, curvature, and length^48^. The network has a high degree of local F-actin alignment (Fig. 1c, Supplementary Figs. 2, 3) like crowded F-actin (Fig. 1b), although is non-uniform in density (Supplementary Fig. 3) and mesh size (Supplementary Figs. 3, 4). By contrast, at 830 nM mDia1, filaments are shorter, and the network is dense, isotropic, and structurally homogeneous on ~1–10 μm length-scales (Fig. 1e bottom, Supplementary Figs. 3–5). In this case, the network grows in the *z*-direction and acquires a thickness of 0.56 μm ± 0.09 μm (Supplementary Fig. 5).

F-actin nucleation by Arp2/3 results in the growth of branched actin networks, whose density and alignment depend upon the concentration of Arp2/3 (Fig. 1d, e top, Supplementary Figs. 2, 3). At 0.74 nM Arp2/3, actin filaments elongate and yield an overall alignment that is comparable to that of 83 nM formin (Fig. 1c, Supplementary Figs. 2, 3)^33,34^. By contrast, at 7.4 nM or 74 nM Arp2/3, the alignment decreases, and the actin grows as clusters which merge into a structurally homogeneous, dense, ‘gel-like’ network (Fig. 1d, e top, Supplementary Figs. 2–5, Supplementary Movie 2)^49^. At the concentration of 74 nM, the network acquires a thickness of 1.63 μm ± 0.47 μm (Supplementary Fig. 5).

### F-actin branching suppresses network contraction

**Fig. 2:**
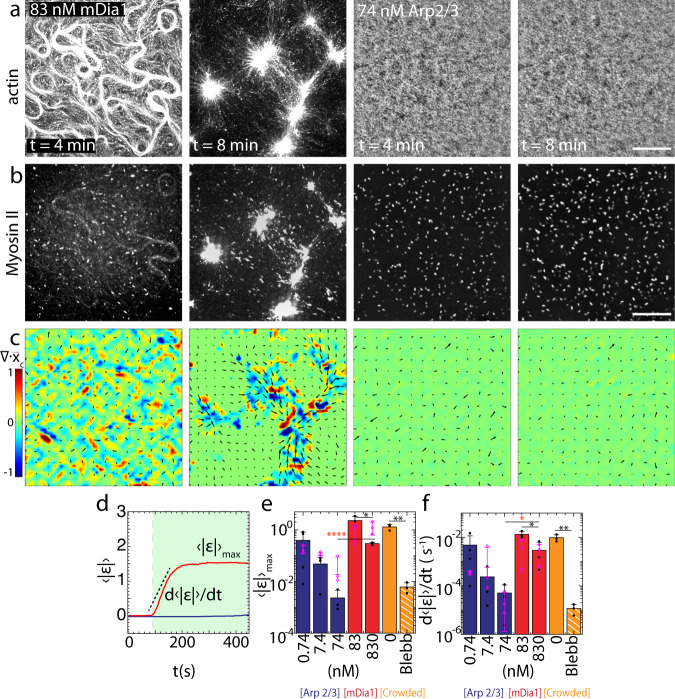
Contractility is attenuated in branched F-actin networks. **a** Fluorescent F-actin over time. *t* is time after the addition of myosin dimers. **b** Fluorescent myosin II on the networks in **a**. **c** Divergence of actin displacements (strain) (colormap) and the local actin velocity field (black arrows). **d** Absolute valued mean strain ($\left\langle {{{{{\rm{\varepsilon }}}}}}\right\rangle$), over time. The strain rate (${{{{{\rm{d}}}}}}\left\langle {{{{{\rm{\varepsilon }}}}}}\right\rangle/{{{{{{\rm{d}}}}}}t}$) and maximum mean strain (${\left\langle {{{{{\rm{\varepsilon }}}}}}\right\rangle }_{{\max }}$) are indicated. Green shaded region indicates the regime of analysis. **e** ${\left\langle {{{{{\rm{|}}}}}}{{{{{\rm{\varepsilon }}}}}}{{{{{\rm{|}}}}}}\right\rangle }_{{\max }}$ for different nucleator concentrations. Blue: Arp 2/3, Red: formin, orange: no nucleator (crowded network), and orange with white stripes: no nucleator +Blebbistatin. (*p < 0.05, **p < 0.01, and ***p < 0.001. *p*(74 nM Arp & 830 nM mDia1)= 0.000081, *p*(83 nM mDia1 & 830 nM mDia1) = 0.029, *p*(no nucleator & no nucleator +blebb)= 0.0023. *N* = 3 independent experiments for each condition. Magenta: no Ni data, *N* = 3 independent experiments for each condition. Error bars represent s.d. of the mean. Two-tailed *t* test.) **f** ${{{{{\rm{d}}}}}}\left\langle {{{{{\rm{|}}}}}}{{{{{\rm{\varepsilon }}}}}}{{{{{\rm{|}}}}}}\right\rangle /{{{{{{\rm{d}}}}}}t}$ for different nucleator concentrations. Color code same as **e**. (*p < 0.05, **p < 0.01, and ***p < 0.001. *p*(74 nM Arp & 830 nM mDia1) = 0.044, *p*(83 nM mDia1 & 830 nM mDia1) = 0.0283, *p*(no nucleator & no nucleator +blebb)= 0.0066. *N* = 3 independent experiments for each condition. Magenta: no Ni, *N* = 3 independent experiments for each condition. Error bars represent s.d. of the mean. Two-tailed *t* test. Source data are provided as a Source Data file).

Skeletal muscle myosin dimers (40 nM) are added to fully assembled F-actin networks and polymerize into thick filaments within the network^46^ (Fig. 2a, b, Supplementary Movie 3–4). Shortly after thick filament assembly, myosin displaces the F-actin (Fig. 2c). The displacement of the F-actin network is quantified by the displacement field (${\vec{{{{{{\bf{x}}}}}}}}_{{{{{{\bf{c}}}}}}}$), as measured from Particle Image Velocimetry (PIV). Thus, we calculate the mean strain $\left\langle {{{{{\rm{\varepsilon }}}}}}\right\rangle$ over time from the divergence of the displacement field, $\left\langle {{{{{\rm{\varepsilon }}}}}}\right\rangle=\left\langle \vec{{{{{{\boldsymbol{\nabla }}}}}}}{{{{{{\boldsymbol{\cdot }}}}}}\vec{{{{{{\bf{x}}}}}}}}_{{{{{{\bf{c}}}}}}}\right\rangle$. If the spatial density of myosin thick filaments *ρ* (#/μm^2^), exceeds a critical concentration *ρ*_*c*_, the accumulation of these stresses results in a net inward, contractile flow ($\left\langle {{{{{\rm{\varepsilon }}}}}}\right\rangle \, < \,0$) within minutes^34^. In this case, there are large-scale deformations as the network is reorganized into asters, as has been reported previously^34,42,50,51^.

From our measurements of ε over time, we extract the maximum mean strain, ${\left\langle {{{{{\rm{\varepsilon }}}}}}\right\rangle }_{{\max }}$, and the strain rate, ${{{{{\rm{d}}}}}}\left\langle {{{{{\rm{\varepsilon }}}}}}\right\rangle /{{{{{{\rm{d}}}}}}t}$ (Fig. 2d, Supplementary Methods). We find that both the maximum strain and the strain rate vary with the type and concentration of F-actin nucleator (Fig. 2e, f). For mDia1-nucleated networks (83 nM and 830 nM), the F-actin is quickly reorganized and compressed, yielding large strains (${\left\langle {{{{{\rm{\varepsilon }}}}}}\right\rangle }_{{\max }} \sim 8{\times }{10}^{-1}\pm 3{\times }{10}^{-1}$ to $1.3{\times }{10}^{-1}\pm 6{\times }{10}^{-2}$) and strain rates (${{{{{\rm{d}}}}}}\left\langle {{{{{\rm{\varepsilon }}}}}}\right\rangle /{{{{{{\rm{d}}}}}}t} \sim {1\times10}^{-3} \pm {3{\times }10}^{-4}$ to $2{\times }{10}^{-3}\pm 1.5{\times }{10}^{-2}$ s^−1^). Within 4 min, the network is contracted from its initial state, either bundled or isotropic, into asters. By contrast, for Arp2/3-nucleated networks (7.4 & 74 nM), and for equivalent myosin thick filament densities ρ, the strains and strain rates are nominal (${\left\langle {{{{{\rm{\varepsilon }}}}}}\right\rangle }_{{\max }} \sim 1.5{\times }{10}^{-1}\pm 4{\times }{10}^{-2}$ to ${1\times10}^{-3}\pm 4{\times }{10}^{-3}$ and ${{{{{\rm{d}}}}}}\left\langle {{{{{\rm{\varepsilon }}}}}}\right\rangle /{{{{{{\rm{d}}}}}}t} \sim 2{\times }{10}^{-3} \pm {10}^{-3}$ to ${1\times10}^{-4}\pm 7{\times }{10}^{-4}$ s^−1^), resulting in minimal F-actin network reorganization.

In nearly all cases, membrane attachment (Ni-HST-bond) attenuates network contractility, consistent with previous results that showed a decrease in contractility with membrane attachment (Fig. 2e, f)^44^. However, it should be noted that in the absence of membrane attachment, the equivalent extent of branching cannot be achieved compared to the cases in which the attachment to the membrane is present (Supplementary Fig. 6). Nonetheless, in the presence or absence of membrane attachment, the decrease in contractility is more pronounced for Arp2/3-nucleated networks than for mDia1-nucleated networks.

Thus far, network contractility follows immediately after myosin assembly within 2 min. However, a decrease in observed contractility may be due to a difference in myosin assembly rates, as thick filaments may assemble at higher rates and therefore contract more quickly on formin-nucleated networks than Arp 2/3-nucleated networks. Therefore, we allow myosin to assemble to completion in the presence of 43 μM of Blebbistatin to inhibit network contractility^52^. Blebbistatin is added to the sample solution prior to myosin filament assembly to inhibit its ATPase activity^53^. Once the myosin filament assembly is complete, Fluorescence Recovery After Photobleaching (FRAP) is performed using 405 nm laser on the sample to inactivate Blebbistatin and to activate the myosin ATPase. As a result of the activation, the F-actin network is contracted. In this case, again, branched actin inhibits contractility in contrast to linear actin (Supplementary Fig. 7, Supplementary Movies 5, 6). Moreover, Heavy Meromyosin (HMM) does not contract branched networks (Supplementary Fig. 8, Supplementary Movies 7, 8).

### F-actin branching prevents the de novo accumulation of mechanical tension

The contractility of networks nucleated by high concentrations Arp2/3 (74 nM) and high concentrations mDia1 (830 nM) are markedly distinct, as the strain varies by over an order of magnitude. Previous work has shown that an increase in connectivity-mediated F-actin network stiffness can reduce network contractility^32^. One potential explanation for this result is that myosin motors have generated mechanical stresses yet are stalled by the generation of large stresses against the increased stiffness of the network. Stalled behavior arises from the accumulation of force and depends upon the load-dependent unbinding rate of myosin^54–56^. However, based on fluctuation analysis and microrheological measurements, we do not detect significant differences in the mechanical properties of networks nucleated by high concentrations of Arp 2/3 (74 nM) or mDia1 (830 nM) (Supplementary Fig. 9)^49,57,58^. Indeed, our measurements of Arp 2/3-nucleated F-actin network elasticity are comparable with previous reports ~1–10 Pa^49,57^. Alternatively, the lack of contractility within Arp 2/3-nucleated networks may indicate that significant myosin-based stresses are not generated. Thus, we infer the accumulation of mechanical stresses by performing laser ablation experiments of actomyosin networks.

**Fig. 3:**
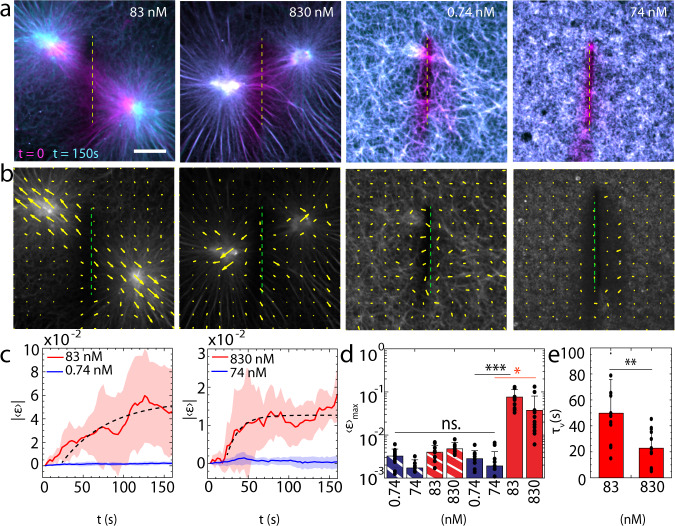
Branched F-actin networks prevent the accumulation of myosin-induced mechanical tension. **a** Actin overlay pre- (magenta, *t* = 0) and post-ablation (cyan, *t* = 150 s). yellow dashed lines show the ablation regions. **b** Actin velocity field quiver plot for post ablation (*t* = 150 s). **c** Absolute valued mean strain ($|\left\langle {{{{{\rm{\varepsilon }}}}}}\right\rangle|$) vs. time for post-ablation (*t* > 15 s) 83 nM mDia1 vs. 0.74 nM Arp 2/3-nucleated F-actin (left), and 830 nM mDia1 vs. 74 nM Arp 2/3-nucleated F-actin (right) (N = 10 independent experiments for each condition). Shaded regions indicate s.d. of the mean. **d** Maximum mean strain (${\left\langle {{{{{\rm{\varepsilon }}}}}}\right\rangle }_{{\max }}$) for actin post-ablation for 83 nM/830 nM mDia1-nucleated F-actin (red), and 0.74 nM/74 nM Arp 2/3-nucleated F-actin (blue). Bars with white stripes = ablation without Myosin II addition. (*p < 0.05, ***p < 0.001, and ns is not significant. *p* (74 nM Arp & 830 nM mDia1) = 0.0206, *p*(0.74 nM Arp & 83 nM mDia1) = 1.66e−5. *N* = 10 independent experiments for each condition. Error bars represent s.d. of the mean. Two tailed *t* test). **e** mDia1 viscoelastic response fit with Kelvin–Voigt model (Methods) (**p < 0.01. *p* = 0.0082. *N* = 10 independent experiments for each condition. Error bars represent s.d. of the mean. Two tailed *t* test. Source data are provided as a Source Data file).

We ablate F-actin networks after myosin addition using an N_2_ pulsed laser (λ = 337 nm, 4 mW) and measure the retraction of F-actin proximal to the location of the ablation (Fig. 3a, b, Methods). The rate of retraction is reflective of the mechanical tension accumulated within the network^59^. Ablation is performed approximately 30 min after the addition of myosin molecular motors. In the case of mDia1-nucleated networks, this is a dynamically arrested state in which connected asters are formed. Ablation is performed along a 20 μm line localized equidistant between the asters, severing the bundles that connect them (Fig. 3a, b, Methods). In the case of Arp2/3-nucleated networks, nominal remodeling of the network has occurred, and thus localization of the line of ablation is arbitrary.

Upon severing the bundles that join asters in mDia1-nucleated networks, the asters retract opposite the region of ablation, indicating the release of mechanical tension (Fig. 3b, c, Supplementary Movies 9, 10). This is expected, as the mechanical force has induced dramatic reorganization of the actin network, through the formation of asters, which slowly merge in time^34,60^. The viscoelastic response of mDia1-nucleated networks fits well with Kelvin-Voigt viscoelastic model (Fig. 3c). Using this model, we identify a viscoelastic timescale, ${\tau }_{\nu }=$ $22\pm 10$s (830 nM mDia1) and ${\tau }_{\nu }=$ $50\pm 25$s (83 nM mDia1), indicating a change in the mechanical properties of the network itself (Fig. 3d, e). Thus, higher concentrations of mDia1 may increase the stiffness of the network. By contrast, Arp2/3-nucleated networks do not retract after ablation, which indicates that no detectable mechanical tension is measurable in the network at this state.

### F-actin branching attenuates the transmission of mechanical stresses

**Fig. 4:**
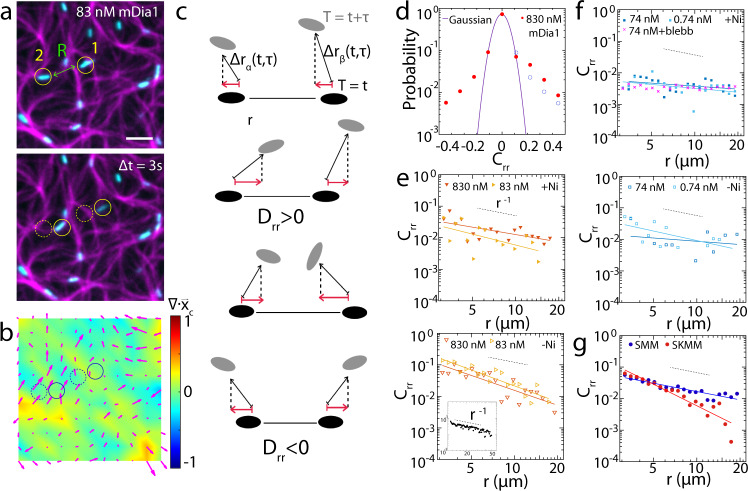
Branched F-actin attenuates the transmission of mechanical strain. **a** F-actin (magenta), Myosin II (cyan) and **b** strain heatmap and local actin velocity quiver plot (magenta) for 83 nM mDia1. Scale bar = 10 μm. **c** Diagrammatic illustration of TPC on Myosin II thick filaments. **d** Probability density distribution for *C*_*rr*_ of myosin thick filaments in 830 nM mDia1-nucleated F-actin network at *r* = 3 μm between −0.5 and 0.5 (red) with Gaussian fit (purple). Blue dashed circles are points mirroring from left half of the plot showing asymmetry for *C*_*rr*_. **e** and **f** show *C*_*rr*_ for myosin thick filaments in mDia1 and Arp 2/3-nucleated F-actin networks over *r* = 3–20 μm. Hollow triangles show data without Ni. Gray dashed lines show slope of −1. Inset: 2 μm beads in glycerol/water (1:3) (black). **g** *C*_*rr*_ for SMM (blue) and SKMM (red) thick filaments in non-nucleated F-actin networks. Gray dashed lines show slope of −1 (*N* = 3 independent experiments for each condition, *N*_pair_ > 1 × 10^6^. Source data are provided as a Source Data file).

To understand the origin of network contractility or the lack thereof, we next monitor the motions of the thick filaments and assess how the material properties of the F-actin network mediates their behaviors. We correlate the motions in time and space at a thick filament density *ρ*, which is below the critical density *ρ*_*c*_, therefore does not yield sufficient stress to induce net contractility on experimental timescales^33^ (Fig. 4a, b, Supplementary Figs. 10, 11, Supplementary Movie 11, Methods). In this state, F-actin experiences filament and network spatial and temporal fluctuations, including local and reversible F-actin ‘plucking’, as has been observed previously^61,62^. These correlations in myosin motion reflect the generation of mechanical stresses by thick filaments that propagate throughout the network and influence the motion of other thick filaments, separated by a distance *r*. To this end, we identify the fluorescence centers of myosin thick filaments (which are ‘pill’-shaped) by Multi-Particle Tracking (MPT). Each myosin thick filament orientation and length are described by the angle and length of the rectangular bounding box enclosing the filament (Supplementary Fig. 12). The positions, angles, and lengths of the geometric centers of the thick filaments are then monitored over time to yield trajectories (Methods). Utilizing position trajectories, we calculate the product of instantaneous velocities, projected onto the direction that joins them, and average over all pairs of thick filaments within the field of view for every lag time τ and pairwise separation *r* (Fig. 4c). The result is a ‘Two-Point’ Correlation (TPC), *D*_*rr*_, as a function of *r*. The correlation is then normalized by the square root of the product of particle speeds to yield the extent to which myosin motions are correlated in space and is termed *C*_*rr*_ (Fig. 4d–g, Supplementary Methods). Using regression, we fit *C*_*rr*_ to a power law of the form, ${C}_{{rr}} \sim 1/{r}^{\alpha }$ (Supplementary Fig. 13). We compare the exponent, *α*, which quantifies the nature of the spatial decay of the correlation. In using this method, we assume that while the network is not contractile on average during the study ($\left\langle {{{{{\rm{\varepsilon }}}}}}\right\rangle \sim 0$), that changes in the fluctuation in time, or the increase in the RMS value of *ε* (Supplementary Fig. 6) does not significantly impact our correlation.

For mDia1-nucleated networks without membrane attachment, we find the correlation in myosin motion decays approximately as ${C}_{{rr}}$ ~$1/r$ (*α* = 0.8±0.2 and 1.15±0.3 for 83 nM and 830 nM mDia1-nucleated F-actin networks respectively) (Fig. 4e). This value indicates that the material may be considered a coarse-grained continuum on the measured length-scales. To test whether this behavior is dependent upon isoform or nucleation condition, we repeat the measurement for non-nucleated and spontaneously polymerized F-actin decorated with smooth muscle myosin (Fig. 4g). Indeed, we find a similar scaling exponent as the conditions in which F-actin is nucleated and using skeletal muscle myosin. Thus, myosin-induced active stresses propagate within entangled networks composed of filamentous actin.

By comparison, the magnitude of the correlation for myosin motions within an Arp2/3-nucleated network is reduced in comparison to mDia1-nucleated networks (Fig. 4f). In this case, the fluctuations are strongly suppressed, and a 1/*r* correlation cannot be assigned. Thus, the propagation of myosin-induced stresses in Arp 2/3-branched F-actin networks is not well quantified. As we cannot measure differences in the mechanics of these networks in the absence of myosin (Supplementary Fig. 3), this may be due to our limits of detection, as the thick filament displacements are too small to emerge as significant in a correlation.

### Branched F-actin inhibits the rotational and translational motion of myosin thick filaments

While the correlation in myosin motions within branched F-actin is reduced, this may be due to decreased actomyosin sliding aside from poor mediation of active stresses by the network. As actomyosin sliding underlies the local production of mechanical stresses, we explore the extent to which sliding, or myosin thick filament processivity is inhibited. To do so, we again identify the positions and angle of myosin thick filaments. From the trajectories, translational and rotational ensemble averaged mean-square displacements (MSD, MSD_θ_) are calculated. In passive systems, the magnitude and exponent of the MSD reflect the geometric and mechanical properties of the environment^63,64^. In active systems, they also reflect the driving force for the probe itself^65,66^.

**Fig. 5:**
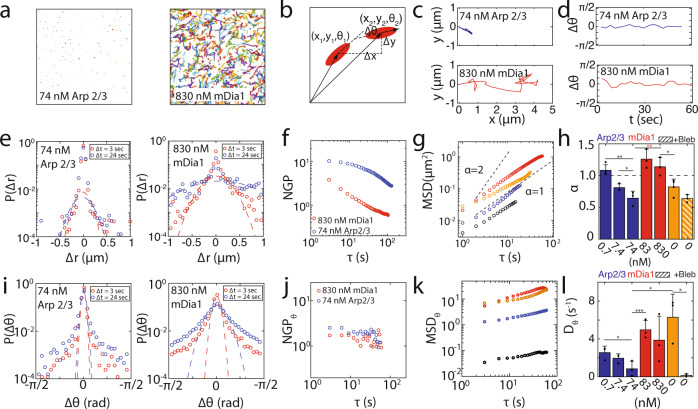
F-actin branching inhibits translational and rotational diffusion of myosin thick filaments. Trajectories of myosin thick filaments over 1 min within **a** 74 nM Arp2/3 and 830 nM mDia1-nucleated F-actin networks. **b** A schematic for measuring translational and rotational motion of a myosin thick filament between two time points. **c**, **d** Individual translational and rotational trajectories of myosin thick filaments. Van Hove relationship **e** and Non-Gaussian Parameter **f** for translational displacement of 74 nM Arp2/3 and 830 nM mDia1-nucleated networks at both 3 and 24 s. **g** Translational Mean-Square Displacement (MSD) of thick filaments. Red: 830 nM mDia1. Orange: Non-nucleated network. Blue: 0.74 nM Arp 2/3. Black: Non-nucleated network with blebbistatin. **h** Slope *α* of the MSD for all conditions. (*p < 0.05, **p < 0.01. *p*(74 nM Arp & 830 mDia1) = 0.0089, *p*(74 nM Arp & 0.74 nM Arp) = 0.002, *p*(830 nM mDia1 & no nucleator) = 0.0248. Error bars represent s.d. of the mean. *N* = 3,3,4,3,3,5,3 independent experiments for the conditions from left to right. Two tailed *t* test). Van Hove **i** and NGP **j** for rotational displacement for 74 nM Arp2/3 and 830 nM mDia1. **k** Rotational MSD for all conditions. Red: 830 nM mDia1. Orange: 83 nM mDia1. Blue: 74 nM Arp 2/3. Black: Non-nucleated network with blebbistatin. **l** Rotational diffusion coefficient of myosin thick filaments for all conditions (*p < 0.05, ***p < 0.001. *p*(74 nM Arp & 0.74 nM Arp) = 0.0499, *p*(74 nM Arp & 83 mDia1) = 0.0051, *p*(74 nM Arp & no nucleator) = 0.0207, *p*(no nucleator & no nucleator +blebb) = 0.0117. Error bars represent s.d. of the mean. *N* = 3,3,4,3,3,5,3 independent experiments for the conditions from left to right. Two tailed *t* test. Source data are provided as a Source Data file).

In observing the translational motion of thick filaments within F-actin networks, we find that over the same time interval (~10 min), that thick filament trajectories are significantly longer in mDia1-nucleated networks than those in Arp2/3-nucleated networks (Fig. 5a, c, Supplementary Movie 12). This suggests that there is an elevated speed of thick filament translational motion in linear networks versus branched networks. As reflected by the translational MSDs, with increasing concentration of either nucleator, the relative magnitude and the scaling exponent *α* of the displacement decreases (Fig. 5g, h). For mDia1-nucleated networks, at low concentrations, the scaling exponent *α* is above 1, or ‘super-diffusive’. Super-diffusive exponents indicate anomalous and directed motion^65^. With further increases in mDia1 concentration, the exponent decreases to 1, indicating diffusive motion. By contrast, for Arp2/3 networks, the exponent is diffusive (*α* = 1) at low concentrations and becomes sub-diffusive (*α* < 1) or ‘caged’ at higher concentrations.

In observing the rotational motion of thick filaments within F-actin networks, we find that angular filament motion also depends on F-actin architecture (Fig. 5b, d, Supplementary Movie 12). The MSD_θ_, is higher in formin-nucleated networks than in Arp2/3 nucleated networks, indicating a higher propensity to rotate in linear compared to branched networks (Fig. 5k). However, in all cases, MSD_θ_ has a modest increase with elapsed time. Still, from the rotational diffusion coefficient D_θ_, we again see an architecture dependence between branched and linear networks (Fig. 5l). Thus, thick filament rotation is highly restricted, suggesting filament motion is principally translational.

The mean displacement of myosin thick filaments may hide ‘rare events’ or small numbers of displacements which are larger or smaller than what is expected by purely random, or diffusive behaviors^67,68^. Thus, despite reduced mean behaviors in the MSD and MSD_θ_, we estimate the prevalence of anomalously small or large displacements in branched and linear networks comparatively. To do so, we look at the distribution of displacements (translational and rotational) using the van Hove function^69,70^ (Fig. 5e, i), and compare to another ensemble metric, the Non-Gaussian Parameter (NGP) (Fig. 5f, j, Supplementary Methods). Indeed, we find that the myosin thick filaments in both mDia1-nucleated networks and Arp 2/3-nucleated networks exhibit a non-Gaussian distribution in fluctuations in displacement and rotation. However, while in mDia1-nucleated networks, myosin thick filaments exhibit anomalously large translational displacements, in Arp 2/3-nucleated networks, the displacements are very small. Therefore, the reduction in motion within Arp2/3-nucleated networks further suggests the properties of the network inhibits the motion of myosin thick filaments.

### F-actin debranching restores myosin thick filament motion

**Fig. 6:**
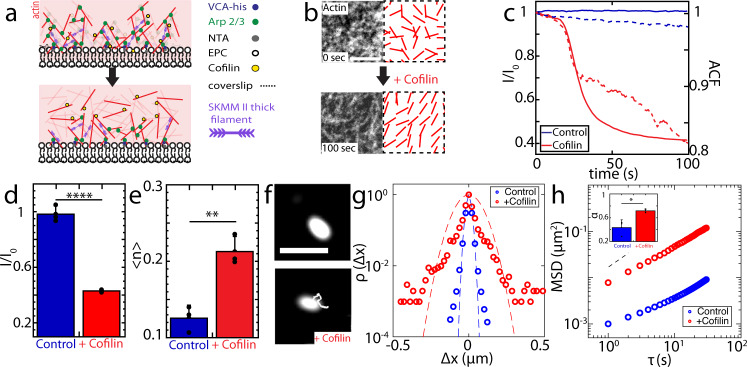
F-actin debranching by cofilin partially restores myosin motility. Step 1: polymerize actin. Step 2: add myosin. Step 3: add Cofilin. **a** Schematic diagram for cofilin debranching process (before: top, after: bottom). **b** 74 nM Arp 2/3-nucleated F-actin network pre- (top) and post- (bottom) addition of cofilin. Right panels show the nematic order alignment. Scale bar = 5 μm. **c** Normalized network fluorescence intensity (*I*/*I*_0_) (left axis) vs. time of the control case (blue solid line) vs. cofilin addition (red solid line), along with myosin autocorrelation (right axis, dashed lines, control: blue, and cofilin addition: red). **d** Steady state actin channel intensity bar plots. (****p < 0.0001. Error bars represent s.d. of the mean. *N* = 3 independent experiments for each condition. Two tailed *t* test). **e** Averaged nematic order parameter <*n*> for 74 nM Arp 2/3-nucleated F-actin network + (red), and -cofilin (blue, control). (**p < 0.01. *p* = 0.0045. Error bars represent s.d. of the mean. *N* = 3 independent experiments for each condition. Two tailed *t* test). **f** Myosin fluorescence & trajectory pre- (top) and post- (bottom) cofilin addition. Scale bar = 2 μm. **g** Van Hove relation of the displacement of myosin thick filaments in 74 nM Arp 2/3-nucleated F-actin network pre- (blue) and post- (red) addition of cofilin at lag time τ = 1 s. Blue and red dashed lines are the Gaussian fits. **h** Ensemble averaged MSD for myosin thick filaments with (red) and without (blue) cofilin addition. Black dashed line indicates slope of 1. Inset: slope of MSD α. (*p < 0.05, *p =* 0.0214. Error bars represent s.d. of the mean. *N* = 3 independent experiments for each condition. Two tailed *t* test. Source data are provided as a Source Data file).

As a control to test the influence of branching on myosin motion, we induce de-branching of the network by the addition of cofilin^71^ (Fig. 6a). To this end, we add 790 nM of Cofilin, to a 74 nM Arp 2/3-nucleated F-actin network, in which myosin thick filaments are assembled and motions are inhibited. The network debranches, as quantified by a decrease in F-actin fluorescence intensity and an increase in the network alignment of F-actin (Fig. 6b–e, Supplementary Fig. 2). As a result, myosin speed increases by 7-fold for short lag times (τ < 15 s, *p* < 0.0001), with a change in the exponent of MSD from 0.35 ± 0.05 to 0.77 ± 0.07 (Fig. 6f–h). Thus, to an extent, the motility of thick filaments can be restored through F-actin debranching, ‘uncaging’ myosin thick filaments at short times. However, ‘caging’ in this context is more complex than in colloidal systems. Debranching of F-actin may lead to decreased geometric confinement, increased F-actin alignment, or a decrease in the number of adhesion sites that limits processive motion^51,72^ (e.g., excess number of crossbridges).

### F-actin architecture determines dependence of myosin speed on thick filament length

To further probe the mechanism by which branched networks inhibit motion, we measure thick filament speed as a function of its size. We hypothesize the potential sources of caging, have different dependences on the length of the filament. For example, increased filament length may yield a greater number of crossbridges at any given time, as well as provide steric hindrance within an F-actin network with small pore size.

**Fig. 7:**
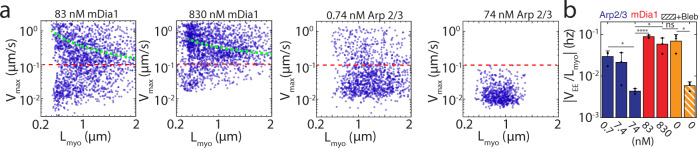
mDia1- and Arp 2/3-nucleated F-actin networks differentially constrain myosin thick filaments. **a** Filament maximum speed (*v*_max_) as a function of myosin thick filament length (*L*_myo_) on networks of different architectures and density. Green dashed curves are ‘guide to eye’ for the inverse relationship. Myosin thick filaments are considered as immobile (pixel resolution = 0.1 μm) below the red dashed lines. **b** Absolute value of the slope of mean speed *v*_EE_ vs *L*_myo_ for all conditions. (*p < 0.05, ****p < 0.0001, and ns is not significant. *p*(74 nM Arp & 0.74 nM Arp) = 0.0148, *p*(74 nM Arp & 83 nM mDia1) = 4.34e-5, *p*(74 nM Arp & 830 nM mDia1) = 0.0172, *p*(no nucleator & no nucleator + blebb) = 0.0229. *N*_myo_ = 3444, 3668, 2258, 2588, 668, 1851, and 775 for each condition from left to right. *N* = 3 independent experiments for each condition. Two tailed *t* test. Error bars represent s.d. of the mean. Source data are provided as a Source Data file).

In all experiments, the size of thick filaments will vary within a field of view. For example, in 74 nM Arp 2/3-nucleated F-actin network, the length of thick filaments ranges from approximately 0.2–1.5 μm, and in 830 nM mDia1-nucleated F-actin network, from 0.2 to 1.8 μm. Thus, for individual experiments, we correlate the translational speeds of myosin filaments (*v*_max_, the maximum speed for each trajectory, and *v*_EE_, the end-to-end trajectory length divided by the total time) with the length of their long axis, *L*_myo_. For mDia1-nucleated networks, we find a modest negative correlation between length and speed (Fig. 7a). Small thick filaments have a greater maximum instantaneous speed than do large thick filaments. However, in comparing the motion *across* concentrations of formin, from 83 nM to 830 nM, minimal changes in speed are observed despite significant changes in network properties, including network pore size (Fig. 7a, Supplementary Fig. 3). Further, in normalizing the speed (over the trajectory) by the thick filament length (${v}_{{{{{{{\rm{EE}}}}}}}}/{L}_{{{{{{{\rm{myo}}}}}}}}$) there is no significant difference between the two conditions (Fig. 7b). At first glance, a size-dependence in the speed within individual experiments may arise from an increase in crossbridges that slow motion or from steric effects. However, as the speed remains the same as network properties change (with formin concentration), the latter is less likely. Thus, in formin-nucleated networks, myosin motion is less restricted by the properties of the network, suggesting the number of crossbridges (which varies with length) is the principal limitation to myosin thick filament motion.

By contrast, for 74 nM Arp2/3, in individual experiments, we find no clear correlation between length and speed for myosin thick filaments (Fig. 7a). Instead, the speed, dramatically reduced in contrast to that in formin-nucleated networks, appears independent of thick filament size. However, in comparing *across* different concentrations of Arp 2/3, we find an overall decrease in speed with increased Arp2/3 concentration. As there is no size dependence in either case, the speed normalized by filament length (${v}_{{{{{{{\rm{EE}}}}}}}}/{L}_{{{{{{{\rm{myo}}}}}}}}$) shows a decrease. The lack of a size-dependence in the speed may be due to locally branched F-actin behaving as a poor substrate for actomyosin sliding. However, steric effects cannot be excluded, as very small pores may inhibit motion across a wide range of thick filament sizes, and thus the influence of geometric confinement may not manifest in an observable size-dependent speed. The pore sizes for 7.4 nM and 74 nM Arp 2/3-nucleated networks are 0.46 ± 0.13 μm^2^ and 0.48 ± 0.03 μm^2^ (Supplementary Fig. 3) as measured by confocal microscopy. Likely an upper bound, based on limits to optical resolution, these length-scales are still smaller than the mean size of myosin thick filaments (Supplementary Fig. 6). Thus, the reduction in myosin speed may be due to a confluence of local geometry (branching) inhospitable to sliding and steric interactions. Separating the influence of steric interactions is not possible in the experiment. Therefore, we seek to understand the roles of each parameter that limits thick filament motion in Arp2/3 nucleated networks using a computational simulation.

### Myosin thick filaments are sterically hindered in branched F-actin

Controlling the effects of steric confinement, F-actin alignment, and crossbridge adhesion experimentally individually are difficult. Therefore, to further explore the impacts of these variables on myosin motion, we utilize agent-based simulation (Methods, Supplementary Methods, Supplementary Fig. 14)^73–75^. By using simulation, we (1) keep constant actin concentration and vary the branching parameter (Arp 2/3 concentration), and (2) keep Arp 2/3 concentration constant and vary myosin thick filament length. However, due to computational limitation, we do not reproduce the initial nucleation and assembly from the surface, but probe the role of steric confinement in mature, fully polymerized networks. As a result, there may be a difference in the net orientation of filaments with respect to the bounding surface, in comparison to experiments. With each condition, we run simulations with and without Volume Exclusion (VE) to represent steric confinement. Volume exclusion accounts for the repulsive physical interactions due to the volume of other objects between physical elements within the simulated network.

**Fig. 8:**
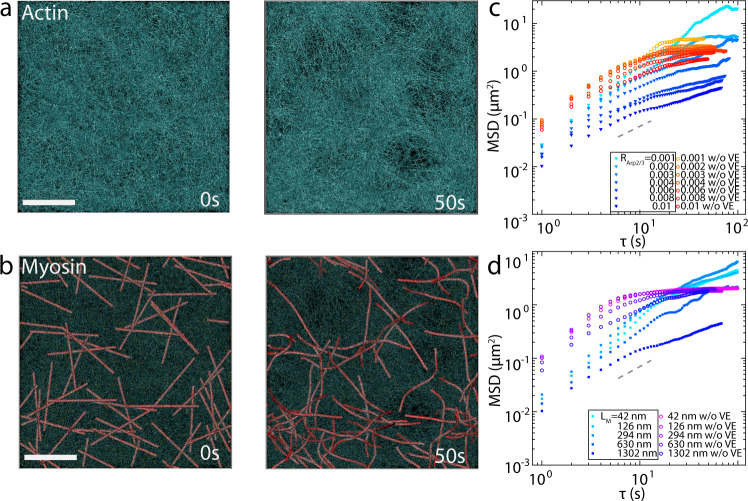
Branched F-actin inhibits myosin motions through volume exclusion. Initial and final state of a network with Arp 2/3-nucleated F-actin (cyan) **a** and actomyosin overlay where motors are shown in red **b** *C*_A_ = 200 μM. *R*_M_ = 0.01. *R*_Arp2/3_ = 0.01. L_M_ = 1302 nm. Scale bar = 1 μm. **c** Ensemble average MSD of myosin with various Arp 2/3: actin ratio (*R*_Arp2/3_) with (solid triangles, boxed legends) and without (hollow circles) volume exclusion (VE). **d** Ensemble average MSD of myosin with various myosin length (*L*_*M*_) with (solid squares, boxed legends) and without (hollow circles) VE. In **c**, and **d**, gray dashed lines indicate slope of 1.

We assemble a densely branched network with F-actin and Arp2/3 (Fig. 8a). After network assembly, myosin motors walk within the network (Fig. 8b), and their MSDs are calculated under various conditions. The simulation is performed under the conditions of the actin concentration (*C*_A_) = 200 µM, the molar ratio of Arp2/3 (*R*_Arp2/3_ = *C*_Arp2/3_/*C*_A_) = 0.01, the molar ratio of myosin (*R*_myo_ = *C*_M_/*C*_A_) = 0.0025, and actin filaments are allocated with biased orientations (Supplementary Table 1). In addition, each myosin motor has 64 arms, corresponding to ~1.3 µm for the equilibrium length of the motor backbone (*L*_M_).

We observe that the motions of motors become more confined as *R*_Arp2/3_ increases (Fig. 8c), which is consistent with our experimental results (Fig. 7b). Comparing the data of the same *R*_Arp2/3_ with and without VE, we see the magnitude of MSD for VE cases is consistently smaller, suggesting the decrease of myosin motion can be due to VE (Fig. 8c, d). While a decrease in *L*_M_ results in much less inhibition of myosin motions (Fig. 8d), an increase in the number of motors has negligible effects on myosin motion inhibition (Supplementary Fig. 15). Meanwhile, a highly branched actin network with many barbed ends pointing outward from the mother filaments can cause difficulties for myosin thick filaments to perform walking or sliding. If only VE plays an important role in motor confinement, the bias in F-actin arrangement would not influence myosin motion. Therefore, we test a simulation with purely random F-actin arrangement. Inhibition in myosin motion is observed (Supplementary Fig. 15), but the degree of inhibition is less than the reference condition with biased F-actin arrangement. This agrees with our experimental data (with and without membrane attachment cases) in which randomly oriented cases (without membrane attachment) show higher contractility (Fig. 2e, f). We find that the F-actin is more densely populated in the lower half of the domain along the *z* direction because the network grows from the bilayer surface (z = 0). The concentrations of actin and Arp2/3 decrease as the network grows in the +*z* direction, resulting in non-uniform density profile. Thus, the local concentrations of F-actin and Arp2/3 in the lower half are found to be higher (Supplementary Fig. 14), whereas the local concentrations in the case with random F-actin arrangement are more uniform. A more uniform density distribution with the same total actin concentration may result in less geometric confinement. To confirm, we run the simulation with random F-actin arrangement and higher *C*_A_ (300 µM). As expected, motor motions are more confined in the case with biased F-actin arrangement. Experimentally, the z-stack image data taken by super resolution microscope shows most of the myosin thick filaments, embedded in the high concentration Arp 2/3-nucleated F-actin network, are restricted at the bottom level (Fig. 8a). This observation matches the simulation result that the biased F-actin arrangement restricts myosin motion, pronounced near the bottom of the network.

Lastly, we explore whether myosin motion can still be restricted to the same extent if myosin thick filaments were not bound to the actin filaments. Three conditions, which are (1) myosin with VE, (2) without binding to F-actin, and (3) without walking, are tested (Supplementary Fig. 15). The no binding case is the highest in magnitude for the MSD due to its diffusive nature. However, as soon as myosin binding is implemented in the simulation, the extent of myosin motion inhibition is similar to when myosin thick filaments are not walking. As a result, we conclude that the cause of such a high degree of confinement of myosin thick filaments by highly branched actin structures could be due to VE effects between cytoskeletal elements when myosin thick filaments bind to F-actin.

**Fig. 9:**
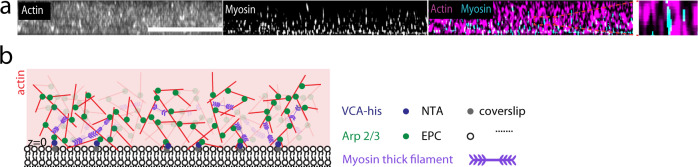
Arp 2/3-nucleated F-actin confines myosin thick filaments. **a** Super-resolution z-stack image for Arp 2/3-nucleated F-actin (left), myosin thick filaments (middle), and merged image of F-actin (magenta) and myosin (cyan). Square image on the right is a zoomed in image of myosin thick filaments embedded in branched F-actin network. Scale bar is 5 μm. The actin (red) and myosin (black) normalized intensity plots as a function of depth z are on the left of actin z stack, respectively. Three independent experiments were done with similar results. **b** Diagram of myosin thick filaments within Arp 2/3-nucleated F-actin network.

Taken together these results illustrate how Arp 2/3-nucleated F-actin can act as a ‘gel’ which encages myosin filaments geometrically to attenuate force generation and transmission (Fig. 9a). Myosin thick filament motions are inhibited by the branched actin structure through local filament organization and geometric confinement which reduces network contractility (Fig. 9b).

## Discussion

The dynamics of network contractility are determined by F-actin network organization. For high concentrations of Arp2/3, F-actin networks are highly branched, and actomyosin contractility is nearly completely attenuated. This stands in partial agreement with previous works that suggest that branching decreases network contractility in the high connectivity regime^39^. In this previous work, myosin VI contracts three-dimensional branched networks, non-monotonically with connectivity. Thus, contractility is restricted at high concentrations, but is promoted at intermediate concentrations. This may be due to differences in the F-actin and branch density, between three-dimensional networks and quasi-two-dimensional networks. Further, myosin VI and myosin II differ in their duty cycle, processivity, walking direction, speed, and filament assembly^76^. Separately, contractility has been observed in Arp2/3 networks coupled to liposome membranes^25^. However, in this case, the growth of actin networks alone accumulates mechanical stress which yields an instability and ultimately breaks symmetry^24^. Myosin activity may nucleate or accelerate this instability over cases in which no myosin is added. In any event, in the work presented here, while branching may aid in percolation above an un-crosslinked network, increased branching unambiguously decreases network contractile strain. Further, in a non-contractile network (ρ < ρ_c_), the motions of myosin thick filaments in Arp 2/3-nucleated networks are poorly correlated, suggesting that F-actin branching suppresses force transmission. However, ablation of actomyosin structures indicates that forces accumulate significantly in mDia1-nuclated networks, but not Arp2/3-nucleated networks. Thus, in addition to reduced force transmission, the de novo accumulation of active stresses is dramatically reduced by F-actin branching.

To account for the differences in contractility, we next assessed the extent to which myosin motion depends upon local F-actin network architecture. Actomyosin sliding generates mechanical forces, and the rate of sliding depends upon the extent of mechanical load^54^. In contractile networks, the extent of sliding is attenuated in comparison to non-contractile networks^33^. Thus, we assess the motion in non-contractile networks, where the number of motors embedded within the network is low. In this case, myosin thick filament motion is reduced in Arp 2/3-branched networks, reminiscent of steric ‘caging’ in passive systems^77,78^. In this case, both translational and rotational motions are inhibited. However, the confining cage presented here is more complex than that observed in passive systems. In addition to steric interactions, there also includes the presence of multiple binding sites that may reduce net motion and local F-actin geometry that is antagonistic to actomyosin sliding. To understand the limitations to the motion of myosin thick filaments, we consider their dependence on thick filament length and how motion varies with changes in network properties, including those that occur with changes in the nucleator used and as well as its concentration.

In formin-nucleated networks, the speed of myosin motion decreases with thick filament length within individual experiments. However, this relationship remains the same across different network conditions, including changes in F-actin alignment and pore size. Thus, these changes in network properties have minimal effects on myosin motion. While the contractility of F-actin networks (bundles) is shown to increase with thick filament size^72^, in non-contractile (unloaded) networks, the motion of myosin thick filaments within an F-actin network may be hindered by increasing potential binding interactions^79^. The observed size-dependence for a single experimental condition therefore, may be due to slowing of the filament with a larger number of crossbridges^80,81^. By contrast, there is no observed size-dependence of myosin filaments in networks with intermediate or high Arp2/3 concentration. However, there is a dramatic decrease in thick filament speed within Arp2/3-nucleated networks in comparison to formin-nucleated networks. Thus, we hypothesize that other mechanisms of inhibition may be dominant within branched networks. Key differences in branched networks in contrast to linear networks include a lack of aligned F-actin suitable for actomyosin sliding as well as potential steric hindrance. Previous studies have shown that a lack of aligned F-actin as ‘tracks’ for myosin does not significantly inhibit myosin motion^33^. However, the lack of F-actin alignment is not equivalent to the presence of F-actin branching. Thus, to further separate these effects, we employ a computational model, where steric effects (volume exclusion) can be decoupled from F-actin network architecture in branched F-actin networks. Using this model, indeed we demonstrate that inhibited processivity is in part due to the local steric confinement.

In summary, we report that Arp2/3-nucleated and formin-nucleated networks are fundamentally distinct in their impact on myosin-induced contractility. Underlying these differences, we suggest that the ‘gel-like’ properties of F-actin networks inhibit myosin thick filament motion through the local geometry and density of F-actin.

In this study, skeletal muscle myosin II (SKMMII) is used, which on average, is longer than non-muscle myosin II (~20 vs. 300 molecules per thick filament respectively). Further, non-muscle myosin II type B (NMM IIB) has higher duty ratio than NMM IIA and SKMM II^82^. Therefore, NMM IIB will also be more processive than SKMMII. However, the density of F-actin within non-muscle cells in vivo, may also be higher than what is accomplished in vitro^83^. In this event, they may still experience restriction from the local density and geometry of F-actin. Thus, these results emphasize the generality of geometric influences on actomyosin contractility^84^.

Recent works that connect the local effects of myosin molecular motors to large-scale fluctuations and contractility consider myosin filaments as idealized force dipoles whose active stresses are nearly uniformly generated, but whose propagation depends upon properties of the fibrous network^85,86^. Here, we show that not all fibrous networks propagate myosin-generated stresses equivalently, and by contrast, in some cases is attenuated nearly completely. At the scale of the filament itself, local confinement effects can inhibit myosin filament motion and therefore myosin filaments cannot be considered ideal force dipoles. Thus, the discrepancy between Arp2/3 mediated network’s connectivity and the lack of contractility lies in its inhibition of the initial generation of active stress. These further highlights contrasting roles of formin-nucleated F-actin and Arp2/3-nucleated F-actin in determining the stability and contractility of the cytoskeleton.

mDia1 and Arp2/3-nucleated networks have opposing effects on the accumulation and transmission of mechanical stresses and ultimately on F-actin network contractility. The origin of these differences can be attributed to local constraints on myosin motion by branched structures. Local confinement of myosin is a mechanism for modulating stability and flow of the actomyosin cytoskeleton.

## Methods

### Growing mDia1 and Arp2/3 networks from the surface

Lipids are made by combining the following lipid/chloroform mixtures and drying under argon gas: 100 μL of 25 mg/mL Egg PC (Chicken egg 840051C, Avanti Polar Lipids), 10 μL of 0.5 mg/mL DHPE (Oregon green 488 DHPE Invitrogen), and 10 μL of 5 mg/mL Nickel lipid (18:1 DGS-NTA(Ni), Avanti Polar Lipids). The lipid film is then resuspended in 5 mL vesicle buffer (100 mM NaCl, 20 mM HEPES, pH 7.3) by vortexing and placed in a bath sonicator approximately 1 h until it becomes transparent to generate small unilamellar vesicles (SUVs). 300 μL of SUV solution is added onto a piranha cleaned glass coverslip within a Chamlide chamber of maximum capacity 600 μL. SUVs are incubated on glass for 10 min and washed away vigorously with 1X F-buffer (50 mM KCl, 1 mM MgCl2, 10 mM Imidazole, 0.2 mM EGTA, pH 8.5) for 6 washes. Histidine tagged mDia1 protein (83 nM and 830 nM) or histidine tagged VCA (30 μM) is freshly diluted in 1X F-buffer pH 8.5 and is incubated on washed lipid bilayers for 10 min and washed gently with 6 washes of 1X F-buffer pH 8.5. In in vitro assays, the pH is typically at 7.5, however, here we used pH 8.5 because we want to eliminate spontaneous polymerization of actin. Therefore, the pH is set to 8.5 to inhibit the polymerization of filaments in the absence of a nucleator^87^. Actin mix (100 μL) is added to 200 μL of chamber volume (containing 1X F-buffer in the absence of ATP). Unless otherwise mentioned, the actin mix contains 1X F-buffer pH 8.5, 0.5 mM ATP, Glucose Oxidase Catalase, glucose to minimize photobleaching, 700nM G-actin (Cytoskeleton, 15% Rhodamine labeled) and 0.25% methylcellulose (14000 MW, Sigma). In addition to this, the mDia1-nucleated F-actin networks contain 2.1 μM profilin (Cytoskeleton), and the Arp2/3-nucleated F-actin networks contain 0.74, 7.4 and 74 μL of Arp 2/3 complex (Cytoskeleton) respectively. The quoted mDia1 and VCA concentration are the concentrations present in the incubating step before washing away unbound protein. Before myosin addition, the actin is polymerized for 1 h at room temperature.

### Super resolution microscope

DeltaVision^TM^ OMX SR microscopy system with structured illumination microscopy (SIM) technique from GE Healthcare. (OMX version: 4.4.9621-10MX system.) A 60x objective lens is used, with 400 ms exposure time, and 286 MHz gain. Each image is 1024 × 1024 pixels, with pixel size 0.08 × 0.08 × 0.125 μm. All the images are taken after the networks reached steady state (>30 min). For more information, visit link: https://www.imsol.co.uk/wp-content/uploads/2019/06/DV-OMX-SR-brochure.pdf.

### Protein purification

SNAP tagged mDia1(FH1C) containing a carboxy-terminal His(6x)-tag was expressed in E.coli (BL21-Codon Plus (DE3)-RP strain). The cells were induced with 500 µM IPTG at 16 °C and purified using Talon® metal affinity resin according to the manufacturer’s instructions. The mammalian SNAP-mDia1 (FH1C)-HIS plasmid was a gift from David Kovar lab.

N-WASP fragment WWA (aa400–501, also called VCA) containing a carboxy-terminal His(6x)-tag was expressed in E.coli (BL21-Codon Plus (DE3)-RP strain). The cells were induced with 1 mM IPTG for 2 h at 37 °C and purified using Ni-NTA resin according to the manufacturer’s instructions, followed by another purification using the size exclusion chromatography. The pET17b KCK-ratWWA-6xHis plasmid was a gift from Cecile Sykes.

### Fluorescent labeling of skeletal muscle myosin and Heavy Meromyosin

Skeletal muscle myosin, or Heavy Meromyosin (rabbit, Cytoskeleton Inc.) is labeled with an Alexa Flour 647 nm C2 Maleimide in reduced conditions. Briefly, myosin is reduced in labeling buffer (50 mM HEPES, 0.5 M KCl, 1 mM EDTA, 10 mM DTT, pH 7.6) then dialyzed in DTT-free labeling buffer overnight. The dialyzed myosin solution is centrifuged to remove insoluble matter and the supernatant is labeled with Alexa Fluor C2 Maleimide, which is added in 5-fold molar excess of myosin. The reaction is performed for 1 h at 4 °C and quenched with the addition of 1 mM DTT. Labeled myosin are collected after running through a desalting column (Pierce, 5 K MWCO, 5 mL). Optical absorption is measured at 280 nm and 647 nm to determine the degree of labeling. This protocol is adapted from Verkohovsky and Borisy^88^.

### Removing non-catalytically active skeletal muscle myosin

Myosin is centrifuged in the presence of polymerized actin in order to isolate only the catalytically active myosin dimers before use. Briefly, actin is polymerized for 1 h at 4 °C in high salt (1X F-Buffer + 4 M KCl) and stabilized with phalloidin. The actin network is spiked with ATP to 1 mM, and freshly thawed myosin is added. The actin-myosin mix is incubated for 10 min and then centrifuged at 4 °C for 30 min at 128360 × *g*. The supernatant is collected, and the concentration of myosin in the supernatant is determined by UV absorption at 647 nm with a known degree of labeling. Myosin is prepared fresh for experiments.

### Photoactivation experiment

F-actin networks are prepared as the procedures mentioned above except that 43 μM of Blebbistatin is mixed with the protein mix. After the network reached steady state, 0.02 μM of myosin dimers are added to the chamber and wait until the completion of myosin thick filament assembly. We then illuminate a 25 μm wide circular region using a 405 nm laser to activate the motors. However, the myosins in the surrounding environment will also be activated due to the exposure to the laser in general. The time interval for one photoactivation cycle is 3 s.

### Calculating nematic order

The nematic order parameter, *n*, is calculated in MATLAB on experimental data. The local F-actin orientation is determined for each director field of small windows of 3.5 μm by 3.5 μm with 80% adjacent overlap. To calculate the local F-actin orientation, each window is Gaussian filtered and transformed into Fourier space using a 2D fast Fourier Transform (FFT). The axis of the least second moment was determined from the second-order central moments of the transformed window, and the angle of the local F-actin orientation is defined as orthogonal to this axis. Next, the local degree of alignment is calculated between adjacent windows within 3 × 3 kernels. The local nematic order is calculated for the central window in each kernel using the modified order parameter equation:

1 $$ < n > =2\left\langle {{\cos }}^{2}\theta -1/2\right\rangle$$

where θ is the difference in F-actin orientation between the central window and the 8 surrounding windows. This process is repeated for all possible 3 × 3 kernels over an image, yielding a nematic director field with defined director magnitude and orientation for each window over an image. Perfect alignment between adjacent regions within an F-actin network results in an order parameter equal to one.

### Calculating strain

Particle image velocimetry (PIV) is applied on the fluorescent actin images in Matlab (mPIV, https://www.mn.uio.no/math/english/people/aca/jks/matpiv/). The extent of contraction is calculated by defining a strain $\varepsilon= < \vec{{{{{{\boldsymbol{\nabla }}}}}}}{{{{{{\boldsymbol{\cdot }}}}}}\vec{{{{{{\boldsymbol{x}}}}}}}}_{{{{{{\boldsymbol{c}}}}}}} >$, as the divergence of the displacement field. Therefore, we can define the maximum strain (*ε*_max_) and strain rate (d*ε*/d*t*) for each network. The data is analyzed with PIV window size 32 and overlap 0.5. Background subtraction from ImageJ is applied to Arp 2/3 contraction data to enhance the Signal-to-noise ratio (SNR).

### Calculating MSD

First, myosin thick filaments are identified by their peaks in fluorescence using ImageJ. The positions are then tracked in time using Crocker-Grier algorithm implemented in MATLAB. Then, the dynamics of myosin thick filament motion is quantified by the mean-squared displacement (MSD). A difference in position within this plane is taken over an elapsed (real) time *t*, between the initial position of a thick filament, $\vec{r}$(0), and the displacement over the elapsed time, $\vec{r}$($t$). MSD can then be defined as

2 $$4{D}_{T}{t}^{\alpha }=\left\langle \frac{1}{N}{\sum }_{i=1}^{N}{(\vec{{{{{{\boldsymbol{r}}}}}}}\left({{{{{\bf{0}}}}}}\right)-{\vec{{{{{{\boldsymbol{r}}}}}}}}_{{{{{{\boldsymbol{i}}}}}}}{{{{{\boldsymbol{(}}}}}}{{{{{\bf{t}}}}}}{{{{{\boldsymbol{)}}}}}})}^{2}\right\rangle$$

Where *D*_*T*_ is the diffusion coefficient and *α* is the exponent of the motion.

Similarly, angular orientation of the filaments is defined as the orientation, θ of their long axis. The angle θ is the angle between the myosin long axis and the *x*–*y* plane (see Supplementary Fig. 13). The mean square change in orientation is then calculated to define a rotational diffusion coefficient, *D*_*θ*_.

3 $$2{D}_{\theta }{t}^{\alpha }=\left\langle \frac{1}{N}{\sum }_{i=1}^{N}{\left(\vec{{{{{{\boldsymbol{\theta }}}}}}}\left({{{{{\bf{0}}}}}}\right)-{\vec{{{{{{\boldsymbol{\theta }}}}}}}}_{{{{{{\boldsymbol{i}}}}}}}\left({{{{{\bf{t}}}}}}\right)\right)}^{2}\right\rangle$$

### Van-Hove analysis

The position of a motor filament is completely defined by its spatial and angular positions, *x*, *y*, and *θ*, respectively. The mean translational and angular step size, ∆*r* and ∆*θ* are then defined as:

4 $$\triangle r=\sqrt{{(x\left(t+1\right)-x(t))}^{2}+{(y\left(t+1\right)-y(t))}^{2}}$$

5 $$\triangle \theta=\theta \left(t+1\right)-\theta (t)$$

Van-Hove function is then defined as the distribution over translational and angular step sizes. For random diffusive motion this distribution should be a gaussian. However, in the presence of anomalous motion (trapped filaments or persistent motion), this distribution deviates from a gaussian.

### Non-Gaussian parameter

From the trajectories of particles, we extract sub-trajectories where the starting and finish point of trajectories are separated by a lag time *τ*. The Non-Gaussian parameter or NGP can then be written in terms of mean 2^nd^ moment $\left\langle {r}^{2}\left(\tau \right)\right\rangle$, and mean 4^th^ moment $\left\langle {r}^{4}\left(\tau \right)\right\rangle$ as

6 $${NGP}\left(\tau \right)=\frac{3\left\langle {r}^{4}\left(\tau \right)\right\rangle }{5{\left\langle {r}^{2}\left(\tau \right)\right\rangle }^{2}}-1$$

NGP is a metric to define the deviation of a distribution from a gaussian. The NGP is bounded below at 0 and at ∞ above, and hence the range is defined as ${NGP}(\tau )\in (0,\infty )$. A NGP of 0 implies that the distribution under consideration is a gaussian. As the magnitude of NGP increases the distribution shifts away from a gaussian distribution.

We include a summary of the metric used to analyze the motion of the myosin filaments:

**Table:** 

Metric	Type	Details
MSD	Ensemble average	Visually representation of average displacement of all filaments over time.
α	Measured over time	Numerical quantification of nature of filament motion. *α* < 1: sub-diffusive; *α* = 1: diffusive; *α* > 1: super-diffusive;
NGP	Ensemble average	Numerical quantification of non-gaussian component of motion. NGP of 0 implies a gaussian distribution.
Van Hove	Instantaneous	Visual representation of type of motion leading to non-gaussian behavior. Trapped filaments show deviation from gaussian distribution around peak, whereas persistent walks show deviation from gaussian at edges.

### Correlation analysis

The autocorrelation analysis is done using our customized MATLAB code. Once the data is loaded as a 3D array (array1), the algorithm finds all the frames that has a delay lag time *τ* for all the frames in array1 and form a 3D array (array2). The mean value for each frame is subtracted from each pixel accordingly for array1 and array2 to only calculate the fluctuations. The product of Image 1 and Image 2 is then the correlation for time lag *τ* between the two frames. For the correlation at specific time lag *τ*, the statistical average is taken for all correlation with time lag *τ* to gain more statistical power. The correlation function is then normalized by the point with lag time dt = 0 (first point of the correlation function). The correlation is then fit to a double exponential:

7 $$a\left(1\right) \,\ast\,{{e}}^{-\left(\frac{t}{a\left(2\right)}\right)}+\left(1-a\left(1\right)\right)\,*\,{{e}}^{-\left(\frac{t}{a\left(3\right)}\right)}+a(4)$$

*a*(1) and (1−*a*(1)) are the coefficients for the two exponentials, and *a*(2) and *a*(3) are the time constants (*τ*) for each exponential. For correlation function fitting we fit over the first (for example 40%) small fraction of the total correlation curve because the points at larger time lag generally not statistically reliable. The larger characteristic time constant is chosen because it reflects, the mechanical property of the network while the smaller time constant can be a result of small actin monomer aggregates’ diffusion, or F-actin serpentation.

### Micro-rheology experiments

We mix unlabeled actin & fluorescent actin mixture (at previously mentioned ratio) with Biotinylated actin monomers (CYTOSKELETON INC, https://www.cytoskeleton.com/actin/ab07) at 10:1 ratio. Fluorescent beads (488 nm fluorescent green) of 0.5 μm in size that are Streptavidin-tagged (BANGS LABORATORIES INC, https://www.bangslabs.com/order-form/CFDG003%7C15557) are mixed into the protein mixture (same conditions as the rest of the experiments in this manuscript) for F- actin polymerization. After full F-actin network polymerization (>30 min after initialization of polymerization), fluorescent beads that are showing thermal fluctuations above the chamber bottom surface (~5 μm from the bottom) are imaged under confocal light microscope at time interval of 0.2 s, ×63 objective, for ~10 min total. There are typically 20–30 particles within the field of view (FOV) (~100 μm × 100 μm).

### Micro-rheology analyses

#### Image processing

All the time series of the beads go through three simple image processing steps: 1. Background subtraction; 2. Bleach correction; 3. Registration. All the steps are done in ImageJ. For the background subtraction, we first apply Gaussian smooth at the width of 0.1 μm, which is the size of one pixel. We then perform background subtraction at the width of the thick filament, 0.5 μm. For bleach correction, we used the ImageJ Plugin: Bleach correction, with the function ‘Simple ratio’, because after the background subtraction, this method shows the highest accuracy with a well estimated background noise level^89^. For registration/de-drift of the time series stack, we used ImageJ’s Plugin: StackReg. We choose the method of ‘translation’ to correct for any translational movements caused by experimental/system uncertainties.

#### Multi-Particle Tracking

Post processing image time series stacks are loaded into IMARIS software (Bitplane). If the background noise is still high, sometimes the background subtraction procedures are performed again in IMARIS. We set the particle tracking particle diameter to be 0.5 μm (same reason as the background subtraction parameter choice). The tolerance for a particle to miss in detection is 3 frames. After that a new ID will be assigned. Particles that have aggregated together are excluded from the data.

#### MSD calculation

The mean squared displacements (MSDs) are calculated using a customized MATLAB script. Data from different experimental runs are combined to calculate the ensemble averaged MSD using Eq. (2). We choose to show 2/3 of the entire data as a function of lag time because the data at larger lag times have fewer points to average, and therefore less reliable.

#### Viscoelasticity (G*) calculation

Customized MATLAB code adapted from Crocker et al.’s IDL code to calculated G(s), G(ω), G’(ω), and G”(ω) using MSD. In practice, two equations are used:

8 $$\alpha \left(\tau \right)=\frac{{{{{{\rm{d}}}}}}\,{{{{{{\rm{ln}}}}}}}\, < \,\Delta {r}^{2}\left(\tau \right) > }{{{{{{\rm{d}}}}}}\,{{{{{{\rm{ln}}}}}}}\tau }$$

9 $${{{{{\rm{|}}}}}}{G}^{*}\left(\omega \right){{{{{\rm{|}}}}}}\approx \frac{{k}_{B}T}{\pi a \, < \,\Delta {r}^{2}(\tau=\frac{1}{\omega }) \, > \,\Gamma [1+\alpha (\tau=\frac{1}{\omega })]}$$

Equation (8) is the logarithmic derivative (slope) of the MSD at particular lag time τ, and Eq. (9) is an approximate, algebraic form of Generalized Stoke–Einstein Relation (GSER)^90^. Result in Eq. (9) assumes that the fluctuations in the network are thermally driven (~${k}_{B}T$), and do not account for polymerization and active components.

### Pore size measurements

One frame of the F-actin in steady state is chosen, and binarized using our customized MATLAB code by having the mean intensity as the cutoff threshold. Each image is chosen to be 750 × 750 pixels (roughly 80 × 80 μm). The output image is analyzed in ImageJ by a Plugin called ‘BoneJ’. The algorithm for the Plugin calculates the area of the dark region surrounded by the white pixels that are connected. The output is then saved and loaded into customized MATLAB code to retrieve the mean pore size and pore distribution information.

### Radial Distribution Function

The image data is binarized with the same fashion as in the pore analysis. Every white pixel is set to be the center of the ring, and the number of white pixels between radius *r* and *r*+*dr* is counted and averaged by the length of the circumference of the ring. This is done across all radii *r* and for all white pixels. The radial distribution *g*(*r*) is then fitted over an exponential in the form of $A{e}^{-(\frac{r}{{{{{{\rm{\lambda }}}}}}})}$, where A is a coefficient (SFig. 3E). The parameter λ is the characteristic length obtained from the exponential fit^91^.

### Two-point correlation

The relationship between the displacements of two myosin thick filaments is:

10 $${D}_{\alpha \beta }\left(r,\,\tau \right)= < \Delta {r}_{\alpha }^{i}\left(t,\,\tau \right)\Delta {r}_{\beta }^{j}\left(t,\,\tau \right)\,\delta [r-{R}^{{ij}}(t)]{ > }_{i\ne j}$$

The two-point correlation (TPC) function, *D*_*rr*_ (see Equation above), typically used for Two Point Microrheology, is used on Myosin II thick filaments to quantify the pairwise correlation^64^. ${R}^{{ij}}(t)$ is the inter particle (in this case, myosin) distance at time t. For example, if we are calculating ${D}_{{rr}}(t,\tau,r)$ at lag time *τ* = 5 s, we are ensemble averaging all pairs at different starting time *t*, but at the same lag time *τ* = 5 s, then ${R}^{{ij}}(t)$ is every pair’s distance at the starting time. *D*_*αβ*_ is the generic form, and here we are interested in the pairwise displacement correlation along the interparticle axis, therefore *D*_*αβ*_ becomes *D*_*rr*_. The correlation function is normalized by the average of the product of displacement from myosin thick filament pair, e.g., *α* and *β*. The code is modified from the original Two Point Correlation code with normalization, along with customized code to combine different runs of the experiments under the same conditions to achieve better statistical significance. Each condition contains over 2000 frames and thousands of thick filaments which result in over millions of pairs to feed into the calculation. We normalize *D*_*rr*_ by using Eq. (11):

11 $${C}_{{rr}}= < \frac{{D}_{{rr}}}{\sqrt{{(d{x}_{\alpha })}^{2}+{(d{y}_{\alpha })}^{2}}\sqrt{{(d{x}_{\beta })}^{2}+{(d{y}_{\beta })}^{2}}} > $$

The normalized *D*_*rr*_ matrix (*C*_*rr*_) is then averaged over the first 10 lag time entries (0.5-1% of the total time) as a function of pairwise-distance r (in μm). The range for power law fitting is chosen to be from 3 μm to 20 μm, which is approximately the range in which the correlation is the most reliable and positive definite. The bin number is set to be 40 (*dr* = range of *r*/bin number).

Myosin thick filaments tracking is done using Imaris (Bitplane). Gaussian blur and background subtraction are first applied to all data within Imaris, and then spot tracking is done within the software using the best parameters that allow the highest accuracy.

### Ablation experiments

The ablation is done using the Micropoint laser setup. The laser (435 nm, Andor Technology) ablates a line shape with 30 μm in length to cut the F-actin network. A 60× oil-immersion objective (Leica Microsystems) was used for ablation and the laser power was held between 60% and 65%.

All confocal microscopy image acquisitions are done using Andor 3.6 software (Andor Technology). The protocol is set to have 5 frames of dt = 3 s pre-ablation, and 50 frames of dt = 3 s post-ablation. The experimental parameters are consistent for all conditions. The data is then cropped around the ablation region into 450×450 pixels (47.7 μm × 47.7 μm). Drift and photobleach during the experiments are eliminated using ImageJ’s plugin called StackReg (Rigid Body) and Bleach Correction (Histogram Matching). The customized MATLAB code with PIV method is used to analyze the cropped data to extract the max mean strain and strain rate data same as how it is done for contraction analysis. The PIV analysis parameters are chosen to have windowsize = 32 and overlap 0.5. The parameters shown in Fig. 3 have windowsize = 64 for better visual illustration.

Mean strain vs. time plots for the mDia1 data are fitted with Kelvin-Voigt model to extract the viscoelasticity characteristic time, and the equation is shown below:

12 $$\epsilon \left(t\right)=\frac{\sigma }{{E}_{v}}(1-{{e}}^{-\frac{t}{{\tau }_{v}}})$$

where $${\tau }_{v}=\frac{{\eta }_{v}}{{E}_{v}}$$.

### Model overview

We employ an agent-based model based on the Langevin equation for Brownian dynamics (Supplementary Fig. 14a–d). The model consists of a branched actin network formed by Arp2/3 with motors. The details and parameters of the model are described in below and Table S1. In the model, actin filament (F-actin), motor, and Arp2/3 are coarse-grained by cylindrical segments. We consider bending, extensional, and torsion forces, as well as a repulsive force to account for volume-exclusion effects between all elements. Seed filaments are created in either a biased or random manner. Arp2/3 permanently binds to a mother filament and leads to the nucleation of a daughter filament, resulting in the formation of a branch. Each arm of motors binds to F-actin, and the motor arm walks toward the barbed end of F-actin and unbinds from F-actin at force-dependent rates.

For all simulations in this study, we employed a rectangular computational domain (4 × 4 × 1.5 μm) with periodic boundary conditions in the *x* and *y* directions. In the *z* direction, the boundaries of the domain exert repulsive forces on elements that are displaced beyond the boundaries. At the beginning of each simulation, a branched actin network is formed via self-assembly of F-actin and Arp2/3. After the network assembly, in cases with biased F-actin arrangement, motors are created near the -z boundary (i.e., z ~ 0 μm), whereas in cases with random F-actin arrangement, they are assembled near the midpoint in the *z* direction (i.e., z ~ 0.75 μm). The motors are allocated in such manners to maximize interactions between the branched network and motors, with consideration of directions toward which the motors tend to walk. After motors start walking, their positions are recorded with a regular time interval to calculate the mean square displacement of motors. For more details about the simulation model, please refer to Supplementary Materials: *Details of the agent-based simulation*.

### Reporting summary

Further information on research design is available in the Nature Portfolio Reporting Summary linked to this article.

## Supplementary information

Supplementary Information

Description of additional Supplementary File

Supplementary movies

Reporting Summary

## Data Availability

Raw data supporting the findings of this manuscript are available from the corresponding authors upon reasonable request. A reporting summary for this Article is available as a Supplementary Information file. Source data are provided with this paper.

## Source data

Source Data

## Code availability

Code supporting the findings of this manuscript are available from the corresponding authors upon reasonable request. A reporting summary for this Article is available as a Supplementary Information file.
