# Supplementary material for: F-actin architecture determines constraints on myosin thick filament motion
Source: Nat Commun. 2022 Nov 16;13:7008. doi: 10.1038/s41467-022-34715-6 (PMC9669029; doi:10.1038/s41467-022-34715-6)
Supplement: Supplementary file 1 — Supplementary Information [file 41467_2022_34715_MOESM1_ESM.pdf]

Supplementary Materials for: F-actin architecture determines constraints on myosin thick filament motion

Camelia G. Muresan[1,2,†], Zachary Gao Sun[2,3,†], Vikrant Yadav[1,2], A. Pasha Tabatabai[1,2], Laura Lanier[1,2], June Hyung Kim[4] , Taeyoon Kim[4], and Michael P. Murrell[1,2,3,*]

[1] Department of Biomedical Engineering, Yale University, 55 Prospect Street, New Haven, Connecticut 06511, USA

[2] Systems Biology Institute, Yale University, 850 West Campus Drive, West Haven, Connecticut 06516, USA

[3] Department of Physics, Yale University, 217 Prospect Street, New Haven, Connecticut 06511, USA

[4] Weldon School of Biomedical Engineering, Purdue University, 206 S. Martin Jischke Drive, West Lafayette, Indiana 47907, USA

[†] indicates equal authorship

*To whom correspondences should be addressed. Email: michael.murrell@yale.edu

Supplementary Methods

Details of the agent-based simulation

Brownian dynamics via the Langevin equation

In the agent-based model, the displacements of the endpoints of all segments are determined by the Langevin equation with inertia neglected:

$$\mathbf{F}_i - \zeta_i \frac{\mathrm{d}\mathbf{r}_i}{\mathrm{d}t} + \mathbf{F}_i^{\mathrm{T}} = 0 \tag{S1}$$

where the subscript i indicates the ith endpoint, $\mathbf{r}_i$ is a position, $\mathbf{F}_i$ is a deterministic force, ζ_i is a drag coefficient, t is time, and $\mathbf{F}_i^{\mathrm{T}}$ is a stochastic force satisfying the fluctuation-dissipation theorem [1]:

$$\left\langle \mathbf{F}_i^{\mathrm{T}}(t)\mathbf{F}_j^{\mathrm{T}}(t) \right\rangle = \frac{2k_{\mathrm{B}}T\zeta_i\delta_{ij}}{\Delta t}\boldsymbol{\delta} \tag{S2}$$

where δ_{ij} is the Kronecker delta, $\boldsymbol{\delta}$ is a second-order tensor, and Δt = 1.15x10^{-5} s is a time step. The drag coefficient, ζ_i, is determined via an approximated form for a cylindrical object [2]:

$$\zeta_i = 3\pi\mu r_{\mathrm{c},i}\frac{3 + 2r_{0,i} / r_{\mathrm{c},i}}{5} \tag{S3}$$

where μ is the viscosity of a surrounding medium, and $r_{0,i}$ and $r_{\mathrm{c},i}$ are the length and diameter of a cylindrical segment connected to the endpoint, respectively. The positions of all the endpoints are updated every time step using the Euler integration scheme:

$$\mathbf{r}_i(t+\Delta t) = \mathbf{r}_i(t) + \frac{\mathrm{d}\mathbf{r}_i}{\mathrm{d}t}\Delta t = \mathbf{r}_i(t) + \frac{1}{\zeta_i}\left(\mathbf{F}_i + \mathbf{F}_i^{\mathrm{T}}\right)\Delta t \tag{S4}$$

The deterministic force, $\mathbf{F}_i$, includes extensional forces maintaining equilibrium lengths, bending/torsion forces maintaining equilibrium angles, and repulsive forces representing volume-exclusion effects between overlapping elements. The extensional, bending, and torsion forces are determined by the harmonic potentials:

$$U_{\mathrm{s}} = \frac{1}{2}\kappa_{\mathrm{s}}\left(r - r_0\right)^2 \quad \text{(S5)}$$

$$U_{\mathrm{b}} = \frac{1}{2}\kappa_{\mathrm{b}}\left(\theta - \theta_0\right)^2 \quad \text{(S6)}$$

$$U_{\mathrm{t}} = \frac{1}{2}\kappa_{\mathrm{t}}\left(\phi - \phi_0\right)^2 \quad \text{(S7)}$$

where κ_{s}, κ_{b}, and κ_{t} are extensional, bending, and torsional stiffnesses, r and r_0 is the instantaneous and equilibrium lengths of cylindrical segments, θ and θ_0 are instantaneous and equilibrium bending angles formed by interconnected segments, and ϕ and ϕ_0 are instantaneous and equilibrium torsion angles.

Simplified geometry and mechanics of cytoskeletal elements

F-actin is comprised of serially connected cylindrical segments with polarity defined by barbed and pointed ends. The equilibrium length of actin segments ($r_{0,\mathrm{A}}$ = 140 nm) is maintained by extensional stiffness ($\kappa_{\mathrm{s,A}}$), and an equilibrium angle formed by two adjacent actin segments ($\theta_{0,\mathrm{A}}$ = 0 rad) is maintained by bending stiffness ($\kappa_{\mathrm{b,A}}$) (Fig. S9B). The reference value of $\kappa_{\mathrm{b,A}}$ corresponds to the persistence length of 9 μm [3].

Arp2/3 consists of two cylindrical segments. The equilibrium length of Arp2/3 arms ($r_{0,\mathrm{Arp2/3}}$ = 23.5 nm) is regulated by extensional stiffness ($\kappa_{\mathrm{s,Arp2/3}}$), and an equilibrium angle formed by two arms of each Arp2/3 ($\theta_{0,\mathrm{Arp2/3c}}$ = 0 rad) is regulated by bending stiffness ($\kappa_{\mathrm{b,Arp2/3c}}$) (Fig.

S9c). The right angle between a mother filament and the arm of Arp2/3 ($\theta_{0,\mathrm{Arp2/3m}}$ = 90˚ = 1.22 rad) at equilibrium is maintained by bending stiffness ($\kappa_{\mathrm{b,Arp2/3m}}$), and the equilibrium angle between a daughter filament and the other arm of Arp2/3 ($\theta_{0,\mathrm{Arp2/3d}}$ = 20˚ = 0.35 rad) is regulated by bending stiffness ($\kappa_{\mathrm{b,Arp2/3d}}$). An equilibrium angle between mother and daughter filaments ($\theta_{0,\mathrm{Arp2/3f}}$ = 70˚ = 1.22 rad) is maintained by additional bending stiffness ($\kappa_{\mathrm{b,Arp2/3f}}$). The mother and daughter filaments are enforced to exist on a single plane by maintaining a zero torsion angle between them ($\phi_{0,\mathrm{Arp2/3}}$ = 0 rad) via stiffness ($\kappa_{\mathrm{t,Arp2/3}}$).

Each motor is modeled to reflect the structure of myosin thick filaments. Each motor has a backbone structure with N_{MA} arms, and each arm represents two myosin heads (N_{H} = 2). Thus, the total number of myosin heads represented by one motor is $N_{\mathrm{MA}}N_{\mathrm{H}}$. The equilibrium length of motor backbone segments ($r_{\mathrm{s,MB}}$ = 42 nm) is maintained by extensional stiffness ($\kappa_{\mathrm{s,MB}}$) (Fig. S9D). Due to this equilibrium length, the total length of each motor with N_{MA} arms becomes $r_{\mathrm{s,MB}}(N_{\mathrm{MA}}/2 – 1)$ nm. An equilibrium angle formed by adjacent backbone segments ($\theta_{0,\mathrm{MB}}$ = 0 rad) is maintained by bending stiffness ($\kappa_{\mathrm{b,MB}}$). The value of $\kappa_{\mathrm{s,MB}}$ is identical to $\kappa_{\mathrm{s,A}}$, whereas the value of $\kappa_{\mathrm{b,MB}}$ is much greater than $\kappa_{\mathrm{b,A}}$. The extension of each motor arm is regulated by the two-spring model with the stiffnesses of transverse ($\kappa_{\mathrm{s,MA1}}$) and longitudinal ($\kappa_{\mathrm{s,MA2}}$) springs. The transverse spring regulates an equilibrium distance ($r_{0,\mathrm{MA1}}$ = 53.5 nm) between the endpoint of a motor backbone and an actin segment where the arm of the motor is bound, and the longitudinal spring maintains a right angle between the motor arm and the actin segment ($r_{0,\mathrm{MA2}}$ = 0 nm).

Calculation of repulsive forces

The repulsive force is represented by the harmonic potential [4]:

$$U_r = \begin{cases} \frac{1}{2}\kappa_r \left(r_{12} - r_{c,12}\right)^2 & \text{if} \quad r_{12} < r_c \\ 0 & \text{if} \quad r_{12} \geq r_c \end{cases} \tag{S8}$$

where κ_r is the strength of the repulsive force, r_{12} is a distance between two neighboring elements, and $r_{c,12}$ is a critical distance below which the repulsive force acts. In most of our previous studies, the repulsive force was considered only between neighboring F-actins for lower computational cost. However, in this current study, we account for repulsive forces between all elements to avoid underestimating the importance of volume-exclusion effects for motor confinement. For the calculation of repulsive forces, each type of element is considered as follows. Actin segments are treated as a cylindrical object whose length and diameter are $r_{0,A}$ and $r_{c,A}$, respectively. Arp2/3 is treated as a sphere whose radius is $r_{0,Arp2/3}$. Motors are treated as a cylindrical object whose length and diameter are $r_{0,MB}$ and $r_{c,MB}$, respectively. The distance between two neighboring elements, r_{12}, is calculated by finding a minimum distance between two linear segments (actin-actin, actin-motor, or motor-motor), between a linear segment and a sphere (actin-Arp2/3 or motor-Arp2/3), or between two spheres (Arp2/3-Arp2/3). The critical distance, $r_{c,12}$, is defined using the radius of cylindrical segments or the radius of spheres. In case of cylindrical segments, the calculated repulsive force is distributed to the two endpoints of the segments as explained in our previous study [5].

Dynamic behaviors of cytoskeletal elements

At the beginning of simulations, actin, Arp2/3, and motor exist as implicit monomers without their own positions. After their first dynamic event, they become explicit elements with x, y, and z positions. The rate of their first dynamic event is calculated using their local concentrations.

In most of simulations with biased F-actin arrangement, the formation of seed F-actin is initiated from a nucleation event at a constant rate, $k_{n,A}$, with the appearance of one cylindrical segment on the bottom boundary of the computational domain (i.e., the "-z" boundary). The z position of the pointed end of the nucleus segment is set to 0. The direction of the barbed end of the nucleus segment is randomly determined within 70˚ from +z direction. Seed F-actins are allowed to move immediately after formation. We checked that our results are dependent hardly on whether or not the pointed ends of seed F-actin filaments are fixed at z = 0 (data not shown). Note that this way is different from how the VCA surface creates a branched network. Due to uncertainties in detailed structures of experimentally created networks, we created an ideal branched network with all barbed ends of F-actin oriented toward the same direction. However, to consider less ideal branched networks, in a few simulations with random F-actin arrangement, the seed F-actins appear in purely random orientations and random positions. The polymerization of actins is simulated by adding cylindrical segments up to 4 μm at a constant rate, $k_{+,A}$. The limit of 4 μm is imposed to prevent actin filaments from self-interacting with themselves in the computational domain of 4×4 μm in x and y directions with the periodic boundary condition. The depolymerization of F-actin is not considered.

Binding of one arm of Arp2/3 to binding sites located on actin segments every 7 nm is considered using the local concentration of Arp2/3 and the intrinsic binding rate constant, $k_{+,Arp2/3}$. Note that 7 nm which is used for spacing between binding sites as in our previous models[5] corresponds approximately to two actin monomers in F-actin. Volume exclusion between Arp2/3's is not considered in our model. After binding, Arp2/3 exists as an explicit element with its own position. At the end of the other free arm of Arp2/3, a nucleation event for F-actin takes place at a constant rate, $k_{n2/3,A}$, by adding one actin segment. The pointed end of the added actin segment is

directly linked to the end of the free arm, and an angle between the actin segment in the mother filament where Arp2/3 was previously bound and the newly added actin segment for the daughter filament is set to 70˚. In addition, when binding, two actin segments and two arms of Arp2/3 lie approximately on the same plane. The polymerization of the new F-actin proceeds in the same manner and at the same rate as those for the seed F-actin. It is assumed that connections between two actin filaments and Arp2/3 are permanent without possibility of unbinding.

Motors are created from the rapid self-assembly of backbone segments. Motor arms located on the endpoints of the backbone segments bind to binding sites on actin segments at a constant rate, $40N_{H}$ s^{-1}. Walking ($k_{w,M}$) and unbinding ($k_{u,M}$) rates of the motor arms are determined by the parallel cluster model to represent the mechanochemical cycle of non-muscle myosin II [6, 7]. The details of implementation and benchmarking of the parallel cluster model in our models were extensively described in our previous study [8]. $k_{w,M}$ and $k_{u,M}$ in the model are lower with a higher applied load, based on an assumption that motors exhibit a catch-bond behavior. The unloaded walking velocity and stall force of motor arms are set to ~450 nm/s and ~4.1 pN, respectively.

Determination of simulation parameters

Due to complexity of our model, there are several parameters that we need to set. Supplementary Table 1 lists the values of parameters that we employed for our simulations. Many of the parameter values are inherited from our previous models[5, 8]. In this study, we use a larger diameter for motor backbone, 100 nm, to reflect the thickness of myosin thick filaments, ~300 nm[9]. The number of motor arms is increased up to 64 corresponding to backbone length of ~1.3 µm, which is close to the length of skeletal myosin thick filaments[9]. De novo nucleation rate and Arp2/3-based nucleation rate of F-actin are set to result in well-branched actin networks to a similar extent to

those in experiments. Actin concentration and Arp2/3 density are close to values used in experimental. By contrast, motor density for simulations is smaller than that employed in experiments to avoid very high computational cost. Note that branched actin networks themselves with given concentration are already a highly dense structure that leads to a large number of neighboring pairs that need to be identified. Values of mechanical and geometrical parameters for Arp2/3 are somewhat arbitrarily set to capture the branch structure and rigidity of Arp2/3 in general.

Supplementary Figures

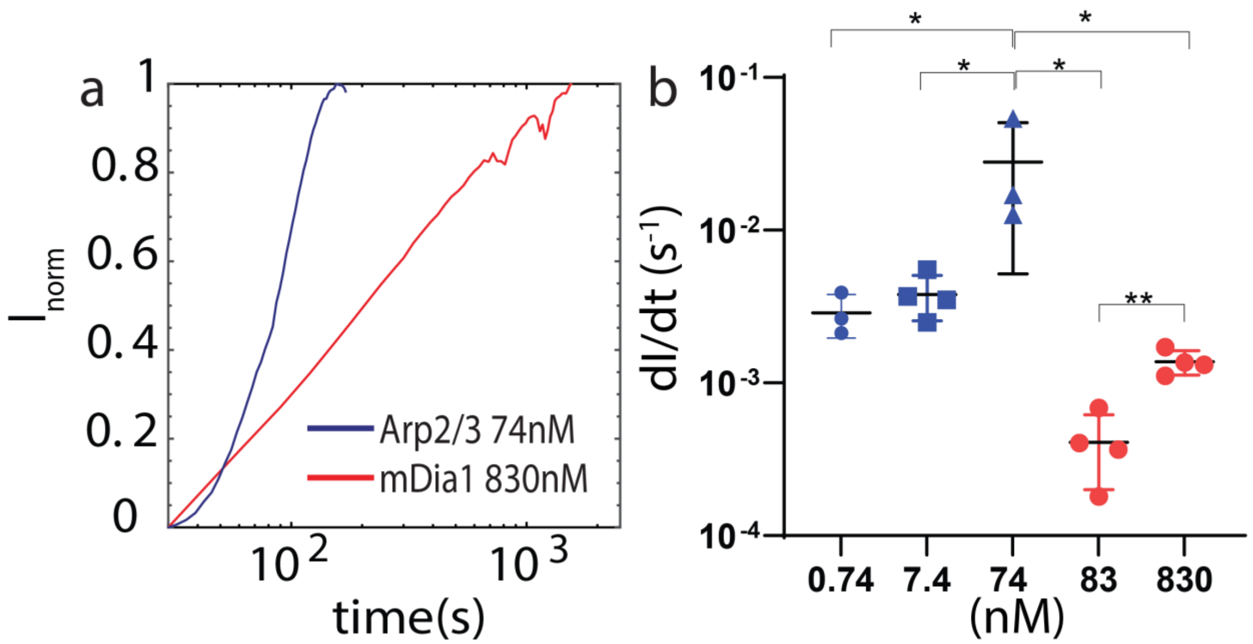

Supplementary Figure 1. Growth of F-actin networks nucleated by Formin mDia1 and Arp2/3. (a) Normalized fluorescence intensity I_{norm} (I/I_0) of actin over time for high concentrations of Arp2/3 and mDia1 bound to the surface of an EPC bilayer. (b) Rate of fluorescence intensity growth (dI/dt) over time for mDia1 (red) and Arp2/3 – nucleated networks (blue) (N = 4 independent experiments for 7.4nM Arp 2/3, 83 nM mDia1, and 830 nM mDia1. N = 3 independent experiments for 0.74 nM and 74 nM Arp 2/3. Error bars are s.d. of the mean. **p*<0.05, ***p*<0.01.)

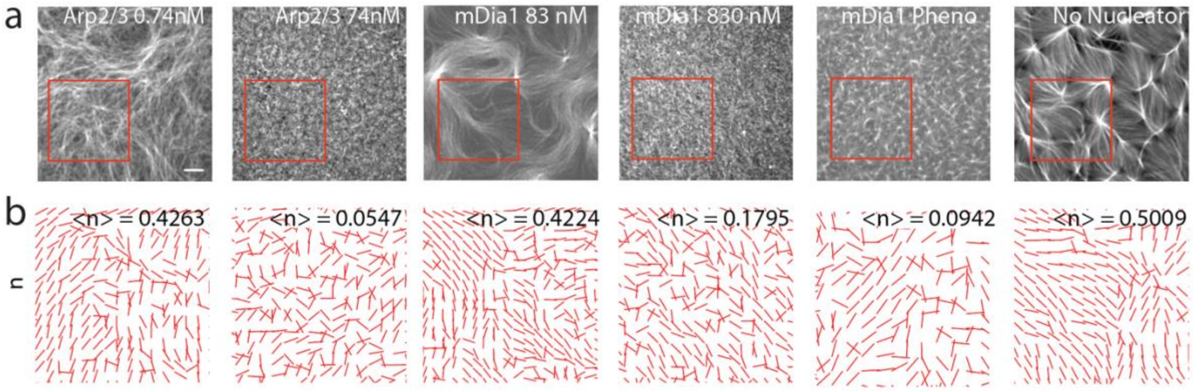

Supplementary Figure 2. Architectures of F-actin networks for all nucleation conditions. (a) Fluorescent actin, for all Arp 2/3 and mDia1 concentrations. (b) Alignment fields (*n)* for all nucleator conditions in (a) with mean values shown. Scale bar is 10 μm.

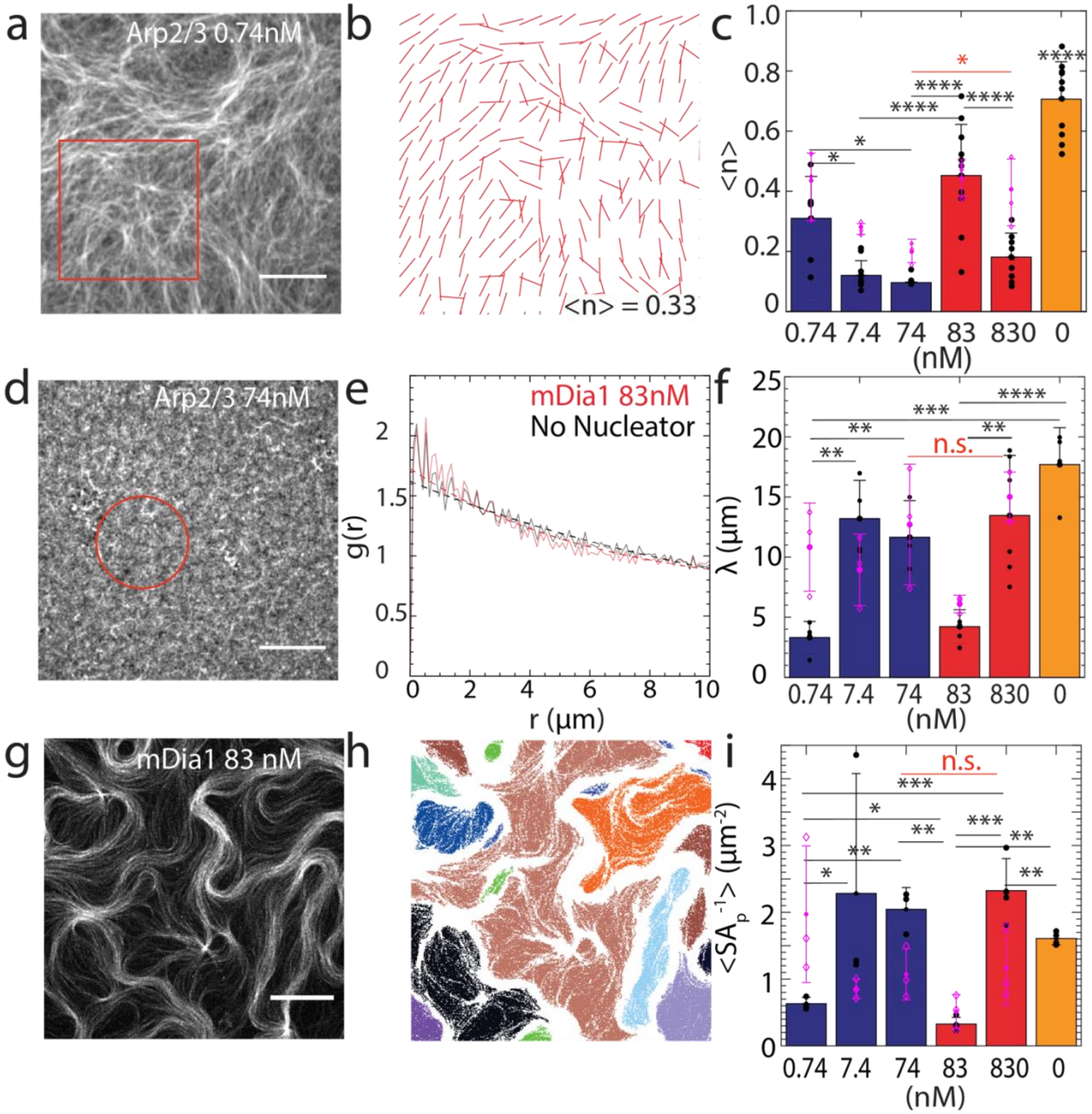

Supplementary Figure 3. F-actin nucleation determines network structure and mechanics. (a) 0.74 nM Arp2/3 network, with red square that outlines region for alignment field (b). Scale bar is 10 μm.(c) Mean order parameter for F-actin alignment ($< n >$) for varying nucleator concentrations. (**p*<0.05, ***p*<0.01, ****p*<0.001, and *****p*<0.0001. N = 8 independent experiments for 830 nm mDia1 and Crowded network. N = 5 independent experiments for each of the other conditions. Magenta: no Ni, N = 3 independent experiments for all conditions. Two tailed *t*-test is performed. Error bars are s.d. of the mean.) (d) 74 nM Arp2/3 F-actin network. Scale bar is 10 μm. (e) Radial distribution function, $g(r)$ for, with exponential fits shown. (f) Exponential length scale (λ) for varying nucleator concentrations (**p* <0.05, ** *p* <0.01, ****p* <0.001, and *****p*<0.0001. N = 3 independent experiments for 7.4 nM Arp 2/3. N = 5 independent experiments

for other conditions. Magenta: no Ni data, N = 3 independent experiments for all conditions. Two tailed *t*-test is performed. Error bars are s.d. of the mean.) (g) 83 nM mDia1 network. Scale bar is 10 μm. (h) Color-coded regions that identify distinct pore sizes. (i) Inverse mean pore area (Sap^{-1}) for varying nucleator conditions. (*p <0.05, ** p <0.01, ***p <0.001, and ****p<0.0001. N = 3 independent experiments for each condition. Magenta: no Ni data, N = 3 independent experiments for each condition. Two tailed *t*-test is performed. Error bars are s.d. of the mean.)

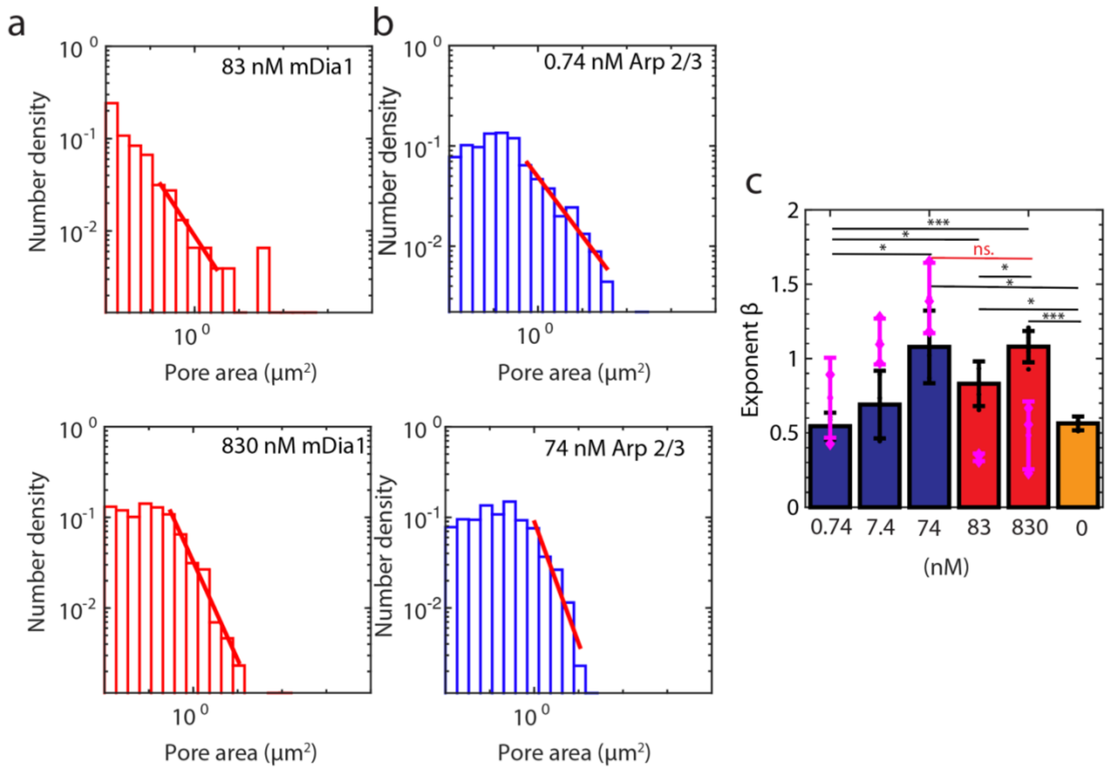

Supplementary Figure 4. F-actin pore size distribution and power law fit exponent. (a) Pore size distribution for 83nM and 830 nM mDia1. Red line is power law fit (b) Pore size distribution for 0.74nM and 74 nM Arp 2/3. (c) Bar plot of power law fit exponent β for all conditions. Cyan = -Ni cases (no adhesion). (*p <0.05, ** p <0.01, ***p <0.001, and ****p<0.0001. N = 3 independent experiments for each condition. Two tailed t-test is performed. Error bars are s.d. of the mean.)

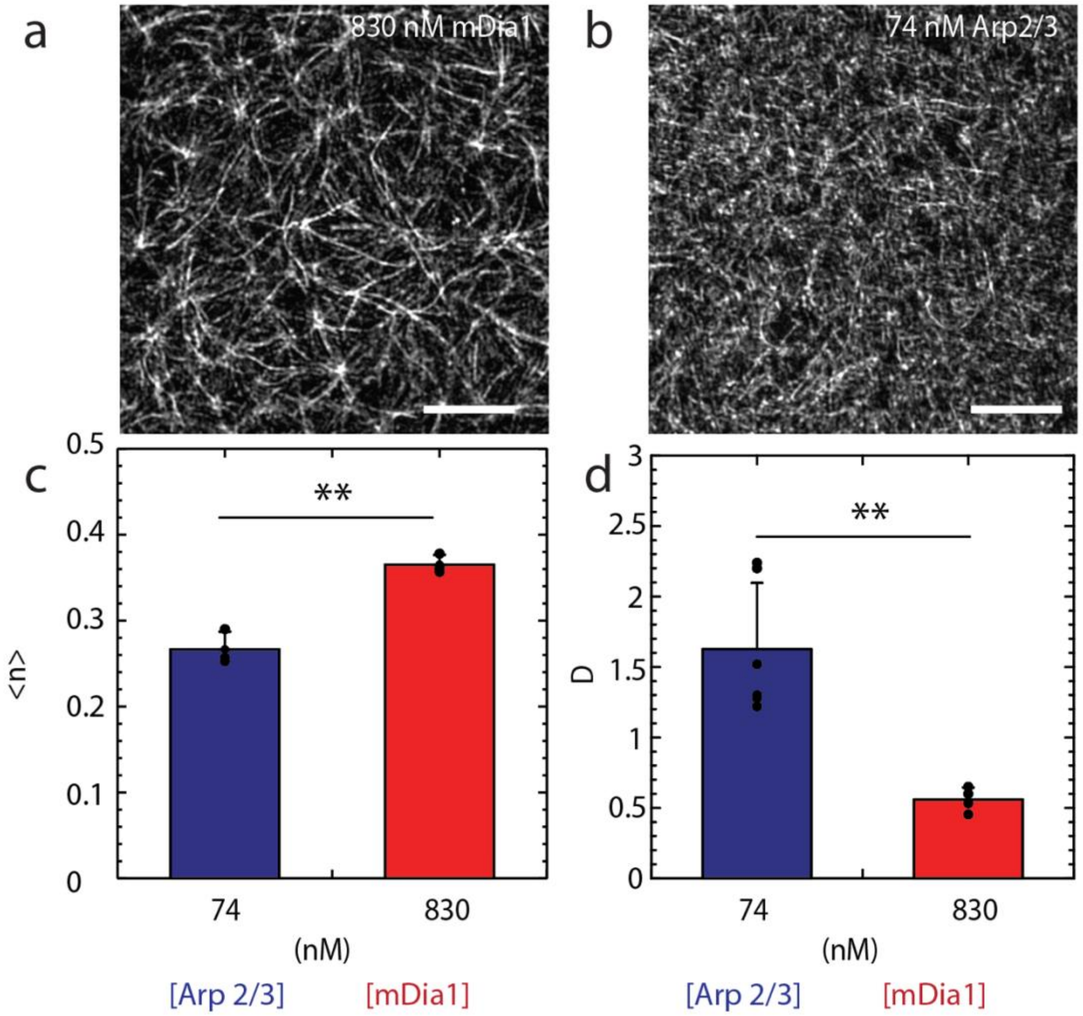

Supplementary Figure 5. Architectures of F-actin networks under super resolution microscope. (a) 830 nM mDia1. Scale bar is 10 μm. (b) 74 nM Arp 2/3. Scale bar is 10 μm. (c) Alignment bar plot for 74 nM Arp 2/3 and 830 nM mDia1 (**p<0.01. N = 3 independent experiments for each condition. Two tailed *t*-test is performed. Error bars are s.d. of the mean.) (d) Thickness bar plot for 74 nM Arp 2/3 and 830 nM mDia1 (***p*<0.01. N = 3 independent experiments, and N = 6 field of view measurements for Arp 2/3, and N = 3 independent experiments, and N = 4 field of view measurements for formin. Two tailed *t*-test is performed. Error bars are s.d. of the mean.)

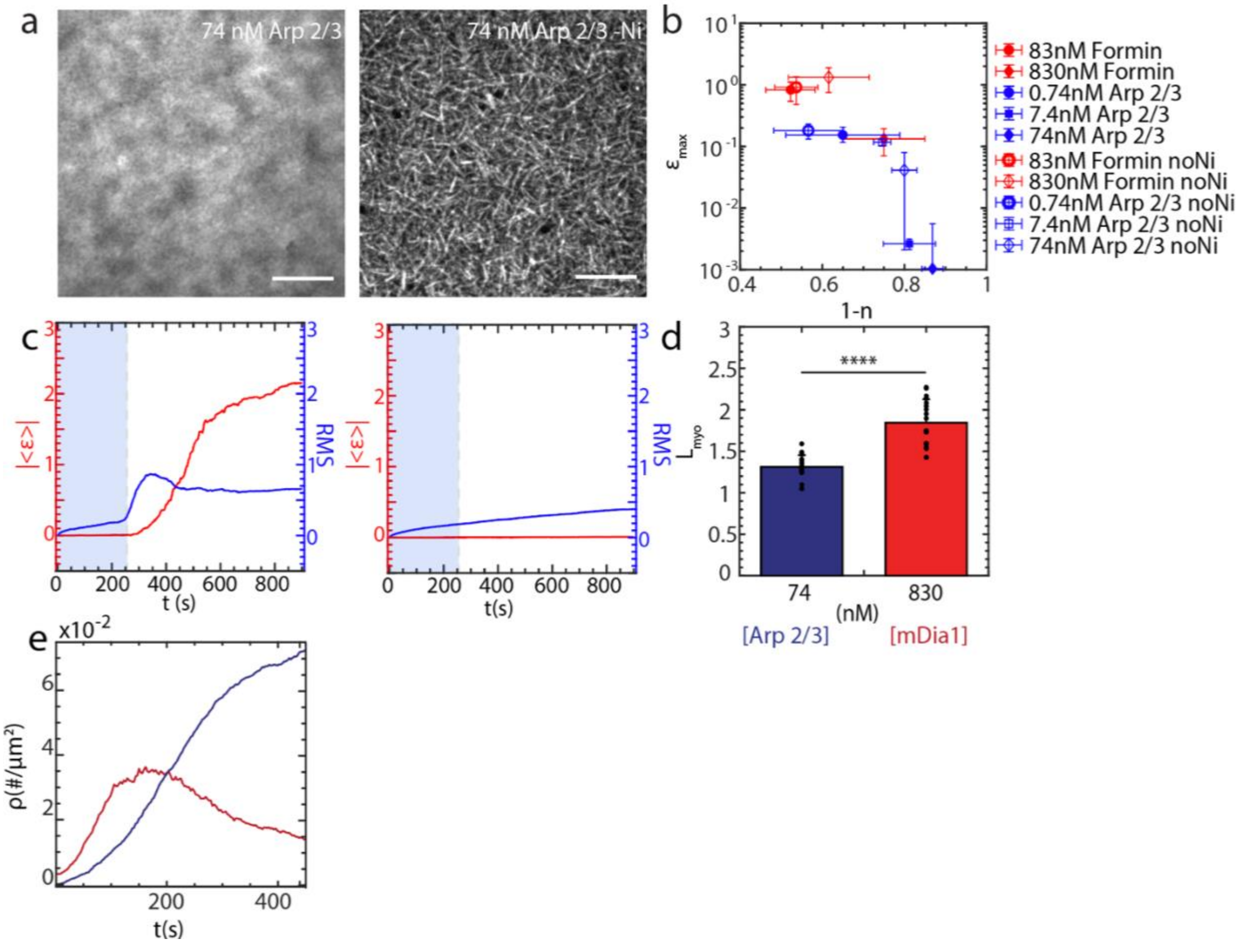

Supplementary Figure 6. F-actin order vs. max strain for all nucleation conditions. (a) Confocal image for 74 nM Arp 2/3 and 74 nM Arp 2/3 -Ni. No adhesion cases result in morphologically and mechanically different structures. Scale bar = 10 μm.(b) F-actin disorder (1-n) vs. mean max strain ($\langle\varepsilon\rangle_{max}$)for all conditions. Solid shapes for +Ni cases, and hollow shapes for -Ni (no adhesion) cases (N = 3 independent experiments for each condition). (c) Network mean strain (red) vs. time. Blue line shows the RMS of the strain. Blue shaded region shows the noncontractile regime ⟨ε⟩= 0, and the rest the contractile regime. Left for 83 nM mDia1 and right for 74 nM Arp 2/3. (d) Myosin thick filament length (L_{myo}) within 74nM Arp 2/3-nucleated F-actin (blue) and 830 nM mDia1-nucleated F-actin (red) (****p<0.0001, N = 20 myosin thick filament measurements for each condition. N = 3 independent experiments. Two tailed t-test is performed with Welch's correction). Error bars are s.d. of the mean. (e) Myosin density, (ρ) over time. Red: 83 nM mDia1. Blue: 74 nM Arp 2/3.

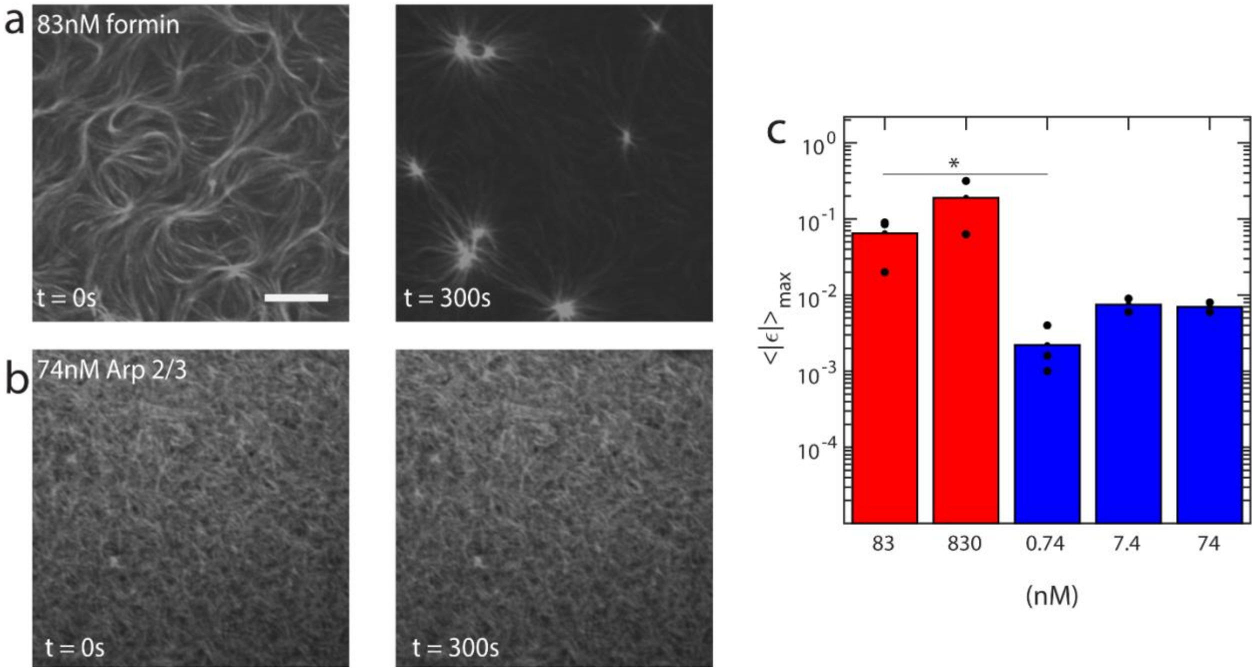

Supplementary Figure 7. Photoactivation experiments. (a) Confocal image for 83 nM formin mDia1 (top) and (b) 74 nM Arp 2/3 (bottom) before and after 300s since 405nm laser activation on myosin thick filaments. Scale bar is 10 μm. (c) Maximum mean strain $\langle|\varepsilon|\rangle_{max}$ for all network types. (*$p<0.05$, N = 3 independent experiments for 83nM formin and 0.74nM Arp 2/3, and N = 2 independent experiments for the rest. Two tailed *t*-test is performed.)

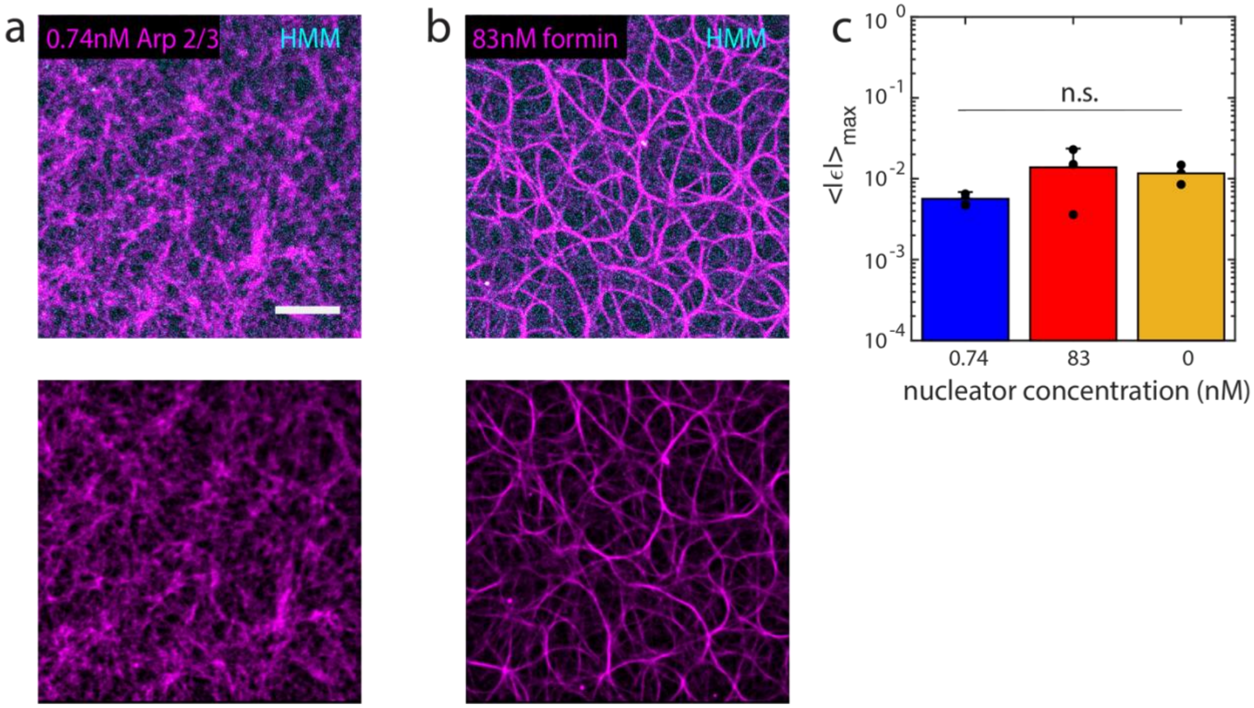

Supplementary Figure 8. HMM experiments. (a) Confocal image overlay for 0.74nM Arp 2/3 and (b) 83nM formin mDia1 nucleated F-actin (magenta) and HMM (cyan). Bottom panels are the actin channel alone. Scale bar is 10 μm. (c) Maximum mean strain $\langle|\varepsilon|\rangle_{max}$ for all network types. (* = p<0.05, N = 3 independent experiments for each condition. Two tailed *t*-test is performed. Error bars are s.d. of the mean.)

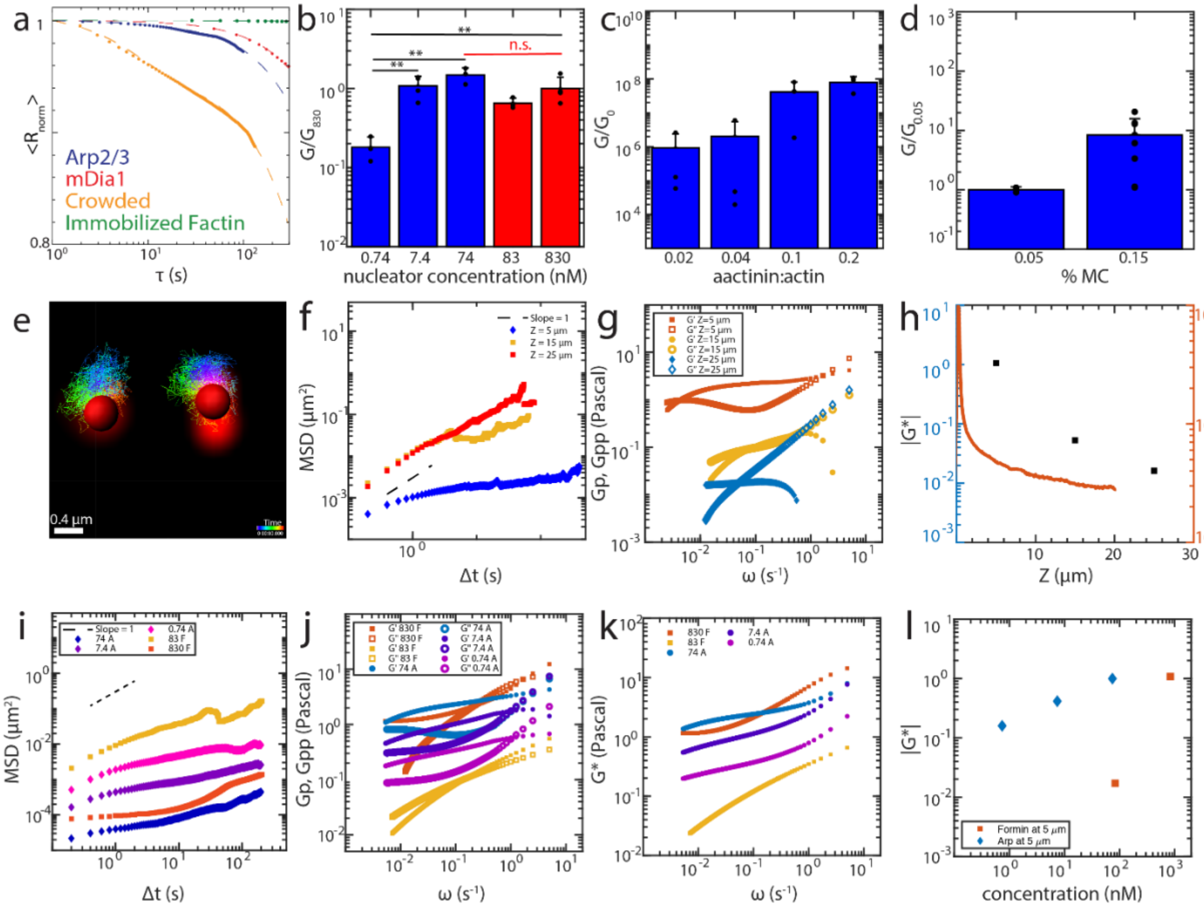

Supplementary Figure 9. F-actin effective stiffness can be inferred using relaxation time. (a) Autocorrelation of image difference. $< R_{norm}(\tau) >=< I(t)I(t+\tau) >/I(0)^2$. (b) G/G_{830}, effective stiffness ratio normalized by 830 nM formin condition (***p*<0.01, ns is not significant. N = 6 independent experiments for 7.4 nM Arp 2/3. N = 8 for Crowded network. N = 3 for all other conditions. Two tailed *t*-test is performed. Error bars are s.d. of the mean.) (c) G/G_0, effective stiffness ratio of α-actinin crosslinked networks with different crosslinker: actin ratio, normalized by crowded network G_0. Error bars are s.d. of the mean. N = 3 independent experiments for all other conditions. (d) Effective stiffness ratio $G/G_{0.05}$, of crowded network under different Methylcellulose concentrations. (N = 2 independent experiments for 0.05 case, and N = 6 for 0.15 case. Error bars are s.d. of the mean.) (e) Streptavidin coated beads trajectory. Red circles show the positions of the two beads. Color code indicates time evolution: from blue to red. Scale bar is 0.4 µm. (f) Ensemble averaged MSDs of beads in the 74nM Arp 2/3 network at different vertical distance (Z) from the bilayer surface. Red: 25 µm, yellow: 15 µm, and blue: 5µm. Black dashed line indicates slope of 1. (N = 2 for each condition, and 2 different field of views of ~20-30 beads for each N.) (g) G' (Storage modulus, closed) and G" (loss modulus, open) of beads in 74 nM Arp 2/3 network at different Z: 25 µm (blue), 15 µm (yellow), and 5µm (red). (h) |G*| at long time scale (large Δt, or small ω) at different Z (black squares, left axis), and F-actin Z- stack normalized intensity (red, right axis). (i) Ensemble averaged MSDs of beads in different types of networks at 5 µm. Black dashed line indicates slope of 1. (N = 2 independent experiments for each condition, and 2 different field of views of ~20-30 beads for each N.) (j) G' (closed) and G" (open) of beads

in different types of networks as a function of frequency (ω). (k) Shear modulus (G^*) for different types of networks as a function of frequency (ω). (l) $|G^*|$ at long time scale (large Δt, or small ω) for different types of networks (blue: Arp 2/3, red: formin).

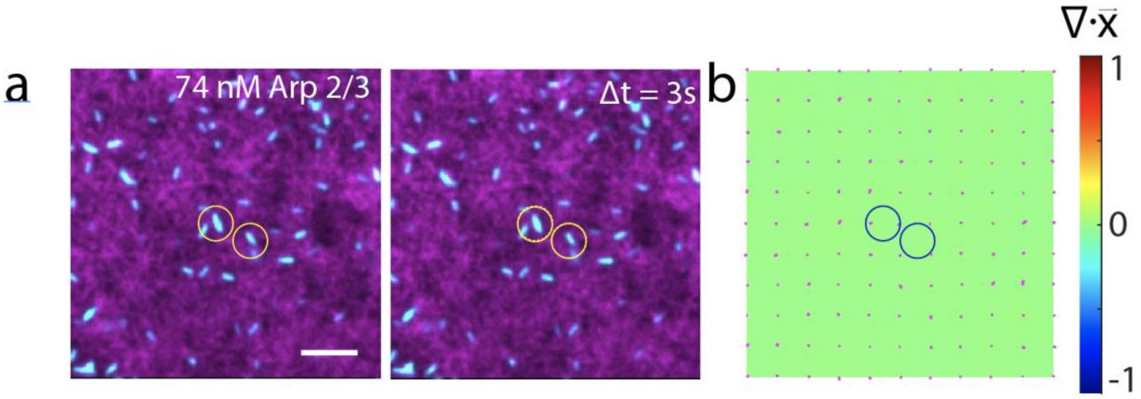

Supplementary Figure 10. Myosin filaments acts as tracer particles for active two point correlation. (a) F-actin (magenta), Myosin II (cyan) and (b) strain heatmap and quiver plot for 74nM Arp 2/3. Scale bar = 10 μm.

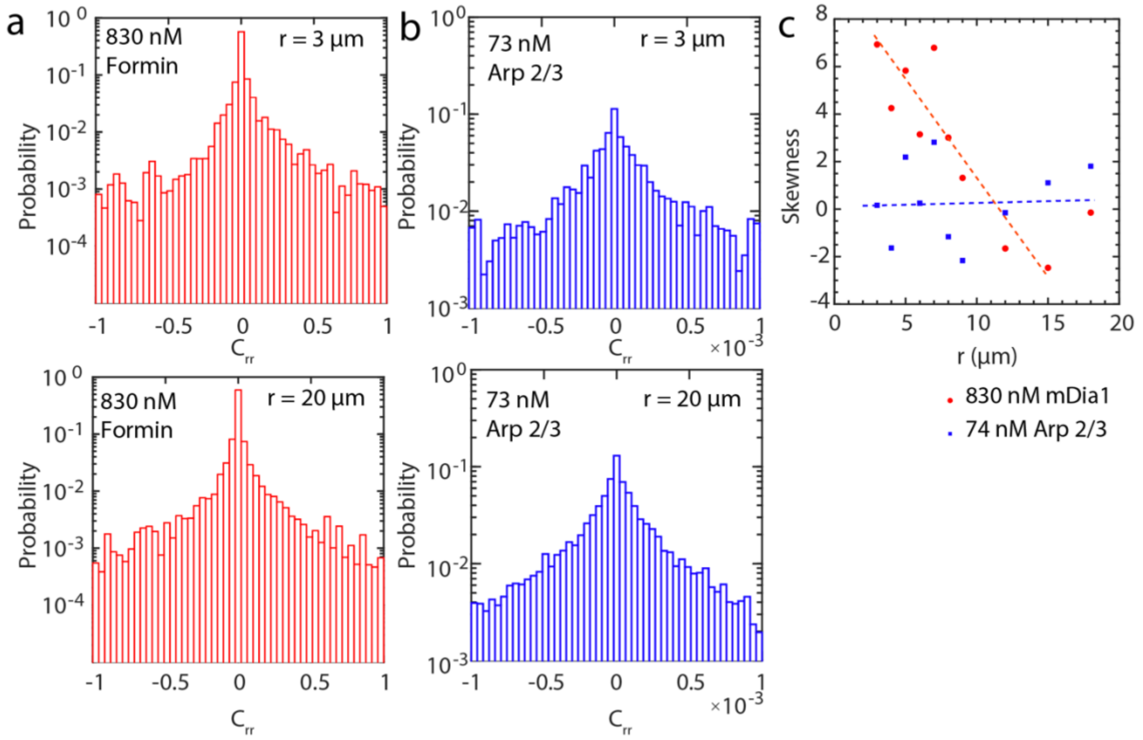

Supplementary Figure 11. Histogram plots of probability distribution for normalized TPC (C_{rr}) (a) Histogram plots for 830 nM mDia1 at r = 3 μm and 20 μm. (b) Histogram plots for 74 nM Arp 2/3 at r = 3 μm and 20 μm. Bin number = 50. Insets show histograms of C_{rr} from interval -1x10^{-3} to 1x10^{-3} (c) Skewness of the histograms at different r values (3 μm < r < 20 μm). Red and blue dashed line is the linear fit to 830 nM mDia1 and 74 nM Arp 2/3 data respectively, slope = -1.168 and 0.025.

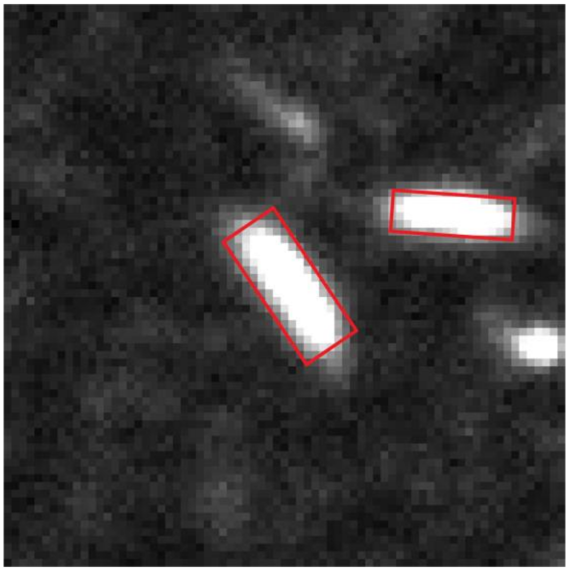

Supplementary Figure 12. Diagram for myosin bounding box. Image showing a minimum spanning bounding box for myosin filaments used for measurements of myosin length and orientation. The box aligns with the axes of the myosin filament body.

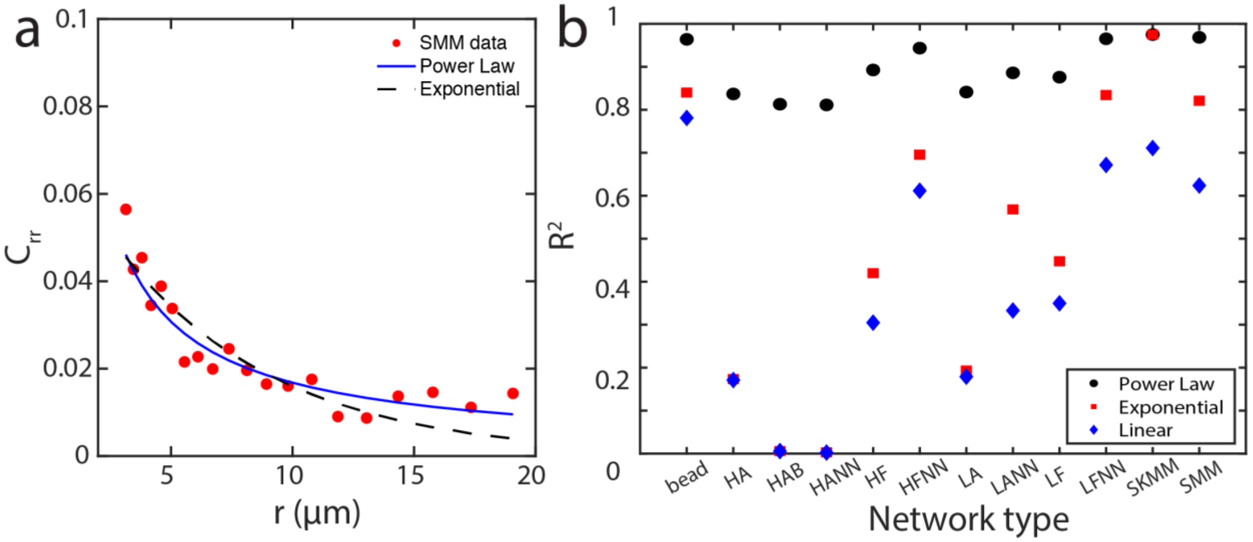

Supplementary Figure 13. C_{rr} **Goodness-of-fit (GOF).** (a) Smooth Muscle Myosin C_{rr} data plotted on linear scale. Red: data, blue line: power law fit, black dashed line: exponential fit. (b) R^2 as goodness-of-fit for all network types. From left to right accordingly: beads in glycerol, 74nM Arp 2/3, 74nM Arp 2/3+blebb, 74nM Arp 2/3 -Ni, 830nM formin, 830nM formin -Ni, 0.74nM Arp 2/3, 0.74nM Arp 2/3 -Ni, Skeletal Muscle Myosin on non-nucleated network, Smooth Muscle Myosin on non-nucleated network. Black circles are for power law, red squares for exponential, and blue diamonds for linear.

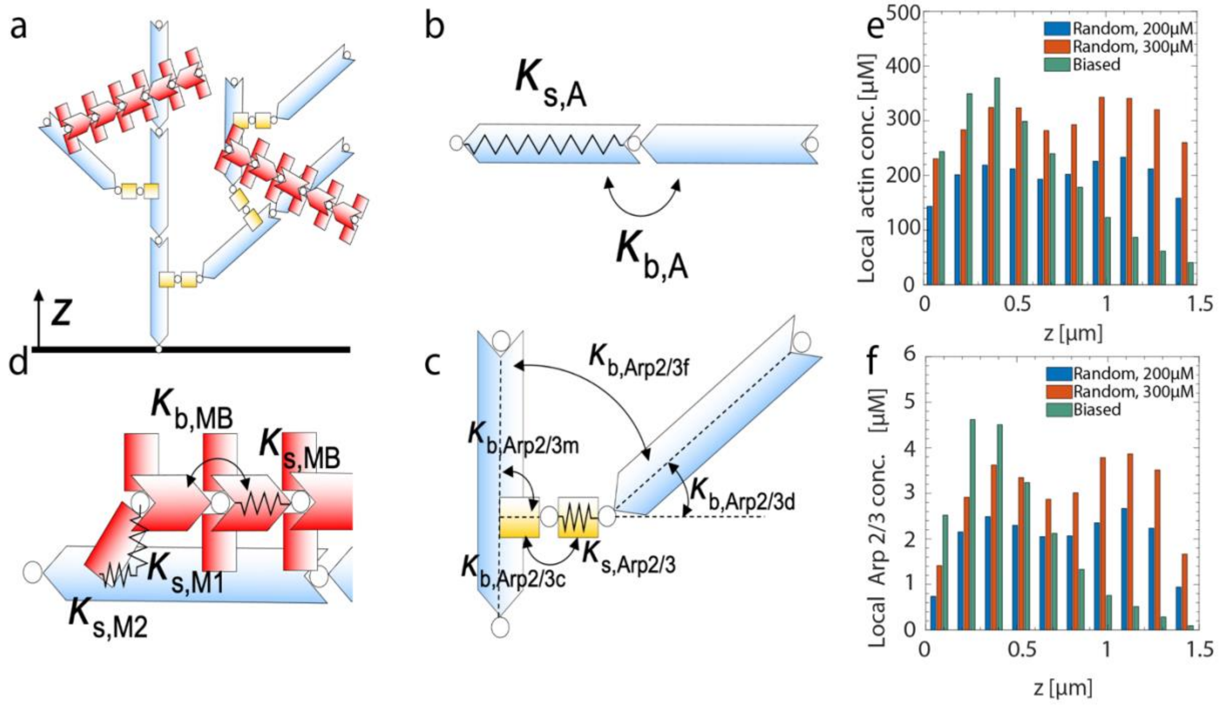

Supplementary Figure 14. Agent-based model for a branched actin network with motors. (a) F-actin (cyan), Arp2/3 (yellow), and motor (red) are simplified by cylindrical segments. In this example, the number of arms in each motor (N_{MA}) is 12. In most cases with biased F-actin arrangement, seed actin filaments are created from the bottom surface (z = 0) with a random orientation within 70˚ from the +z direction, and branches are made by Arp2/3 and daughter F-actins. Motors are created at z = 0, and they tend move in the +z direction due to the bias in the F-actin orientations. In the other cases with random F-actin arrangement, seed actin filaments are created with random orientations and positions, and motors are created at z = 0.75 um. (b-d) Various bending (κ_{b}) and extensional (κ_{s}) stiffnesses maintain lengths and angles near their equilibrium values. The stiffnesses, equilibrium lengths, and equilibrium angles are listed in detail in Table S1. (e, f) Local concentration profiles of actin and Arp 2/3 along the z direction for initial random F-actin arrangement with C_{A} = 200 μM (blue) and 300 μM (red) and for biased F-actin arrangement with C_{A} = 200 μM (green).

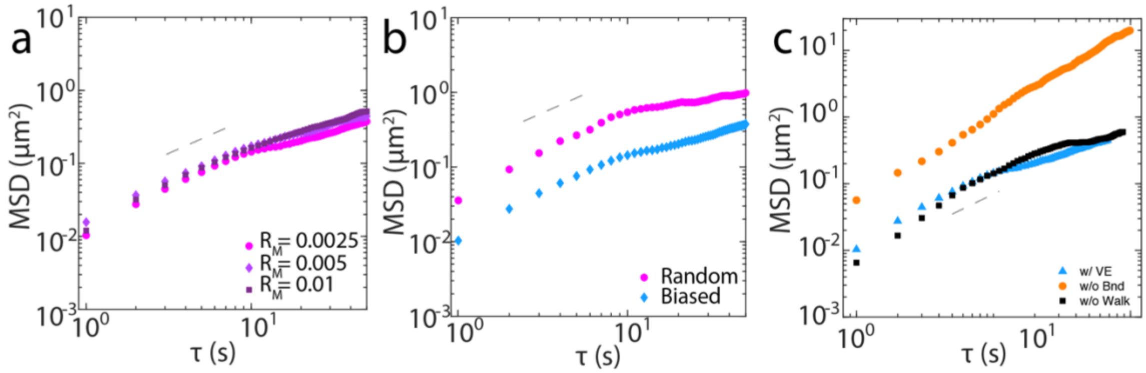

Supplementary Figure 15. Additional MSD plots for different conditions from simulation. (a) Ensemble averaged MSD of myosin for different actin:myosin (R_M) ratios. All dashed grey line show slope of 1. (b) Ensemble averaged MSD of myosin for random (magenta) and biased (cyan) initial actin seed filaments orientations. (c) Ensemble averaged MSD of myosin with VE (blue), without walking (black), and without binding to actin filaments (orange).

Supplementary Table 1. List of parameters employed in the model. For some of the parameters, references are provided if the parameters were determined based on specific previous studies.

Symbol	Definition	Value
$r_{0,\mathrm{A}}$	Length of an actin segment	1.4×10^{-7} [m]
$r_{c,\mathrm{A}}$	Diameter of an actin segment	7.0×10^{-9} [m] [10]
$\theta_{0,\mathrm{A}}$	Bending angle formed by adjacent actin segments	0 [rad]
$\kappa_{s,\mathrm{A}}$	Extensional stiffness of F-actin	1.69×10^{-2} [N/m][8]
$\kappa_{b,\mathrm{A}}$	Bending stiffness of F-actin	2.64×10^{-19} [N·m] [3]
$r_{0,\mathrm{Arp2/3}}$	Length of an Arp2/3 arm	2.35×10^{-8} [m] [11]
$r_{c,\mathrm{Arp2/3}}$	Diameter of an Arp2/3 arm	2.0×10^{-8} [m]
$\theta_{0,\mathrm{Arp2/3c}}$	Bending angle formed by two Arp2/3 arms	0 [rad]
$\theta_{0,\mathrm{Arp2/3m}}$	Bending angle between Arp2/3 arm and a mother filament	1.57 [rad]
$\theta_{0,\mathrm{Arp2/3d}}$	Bending angle between Arp2/3 arm and a daughter filament	0.35 [rad]
$\theta_{0,\mathrm{Arp2/3f}}$	Bending angle between mother and daughter filaments	1.22 [rad][12]
$\phi_{0,\mathrm{Arp2/3}}$	Torsion angle between mother and daughter filaments	0 [rad]
$\kappa_{s,\mathrm{Arp2/3}}$	Extensional stiffness of Arp2/3 arms	2.0×10^{-3} [N/m]
$\kappa_{b,\mathrm{Arp2/3c}}$	Bending stiffness for $\theta_{\mathrm{Arp2/3c}}$	1.0×10^{-19} [N·m]
$\kappa_{b,\mathrm{Arp2/3m}}$	Bending stiffness for $\theta_{\mathrm{Arp2/3m}}$	1.0×10^{-18} [N·m]
$\kappa_{b,\mathrm{Arp2/3d}}$	Bending stiffness for $\theta_{\mathrm{Arp2/3d}}$	1.0×10^{-18} [N·m]
$\kappa_{b,\mathrm{Arp2/3f}}$	Bending stiffness for $\theta_{\mathrm{Arp2/3f}}$	1.0×10^{-18} [N·m]
$\kappa_{t,\mathrm{Arp2/3}}$	Torsion stiffness of Arp2/3	1.0×10^{-18} [N·m]
$r_{0,\mathrm{MB}}$	Length of a motor backbone segment	4.2×10^{-8} [m][8]
$r_{c,\mathrm{MB}}$	Diameter of a motor backbone segment	5.0×10^{-8} [m]
$\theta_{0,\mathrm{MB}}$	Bending angle formed by motor backbone segments	0 [rad]
$\kappa_{s,\mathrm{MB}}$	Extensional stiffness of a motor backbone	1.69×10^{-2} [N/m][8]
$\kappa_{b,\mathrm{MB}}$	Bending stiffness of a motor backbone	5.07×10^{-18} [N·m][8]
$r_{0,\mathrm{MA1}}$	Equilibrium length 1 of a motor arm	5.35×10^{-8} [m]
$r_{0,\mathrm{MA2}}$	Equilibrium length 2 of a motor arm	0 [m]
$r_{c,\mathrm{MA}}$	Diameter of a motor arm	1.0×10^{-8} [m]
$\kappa_{s,\mathrm{MA1}}$	Extensional stiffness 1 of a motor arm	1.0×10^{-3} [N/m][8]
$\kappa_{s,\mathrm{MA2}}$	Extensional stiffness 2 of a motor arm	1.0×10^{-3} [N/m][8]
N_{H}	Number of heads represented by a motor arm	2

N_{MA}	Number of arms per motor	4-64
$k_{n,A}$	De novo nucleation rate of seed F-actin	10^{-5} [μ$M^{-1}s^{-1}$]
$k_{n2/3,A}$	Arp2/3-based nucleation rate of F-actin	10 [μ$M^{-1}s^{-1}$]
$k_{+,A}$	Polymerization rate of F-actin	6 [μ$M^{-1}s^{-1}$][13]
κ_r	Strength of repulsive force	1.69×10^{-3} [N/m]
Δt	Time step	1.15×10^{-5} [s]
μ	Viscosity of surrounding medium	8.6×10^{-1} [kg/m·s]
k_BT	Thermal energy	4.142×10^{-21} [J]
C_A	Actin concentration	200 or 300 [μM]
R_M	Ratio of motor concentration to C_A	0.0025-0.01
$R_{Arp2/3}$	Ratio of Arp2/3 concentration to C_A	0.001-0.01

References

1. Underhill PT, Doyle PS. On the coarse-graining of polymers into bead-spring chains. *Journal of Non-Newtonian Fluid Mechanics* **122**, 3-31 (2004).

2. Clift R, Grace JR, Weber ME. *Bubbles, drops, and particles*. Courier Corporation (2005).

3. Isambert H, *et al.* Flexibility of actin filaments derived from thermal fluctuations. Effect of bound nucleotide, phalloidin, and muscle regulatory proteins. *The Journal of biological chemistry* **270**, 11437-11444 (1995).

4. Kim T, Hwang W, Lee H, Kamm RD. Computational analysis of viscoelastic properties of crosslinked actin networks. *PLoS computational biology* **5**, e1000439 (2009).

5. Jung W, P Murrell M, Kim T. F-actin cross-linking enhances the stability of force generation in disordered actomyosin networks. *Comput Part Mech* **2**, 317-327 (2015).

6. Erdmann T, Albert PJ, Schwarz US. Stochastic dynamics of small ensembles of non-processive molecular motors: the parallel cluster model. *The Journal of chemical physics* **139**, 175104 (2013).

7. Erdmann T, Schwarz US. Stochastic force generation by small ensembles of myosin II motors. *Physical review letters* **108**, 188101 (2012).

8. Kim T. Determinants of contractile forces generated in disorganized actomyosin bundles. *Biomech Model Mechanobiol* **14**, 345-355 (2015).

9. Al-Khayat HA. Three-dimensional structure of the human myosin thick filament: clinical implications. *Global Cardiology Science and Practice* **2013**, 36 (2013).

10. Kishino A, Yanagida T. Force measurements by micromanipulation of a single actin filament by glass needles. *Nature* **334**, 74-76 (1988).

11. Meyer RK, Aebi U. Bundling of actin filaments by alpha-actinin depends on its molecular length. *The Journal of cell biology* **110**, 2013-2024 (1990).

12. Amann KJ, Pollard TD. The Arp2/3 complex nucleates actin filament branches from the sides of pre-existing filaments. *Nat Cell Biol* **3**, 306-310 (2001).

13. Kuhn JR, Pollard TD. Real-time measurements of actin filament polymerization by total internal reflection fluorescence microscopy. *Biophysical journal* **88**, 1387-1402 (2005).
