# Supplementary material for: F-actin architecture determines constraints on myosin thick filament motion
Source: Nat Commun. 2022 Nov 16;13:7008. doi: 10.1038/s41467-022-34715-6 (PMC9669029; doi:10.1038/s41467-022-34715-6)
Supplement: Supplementary file 2 — Description of additional Supplementary File [file 41467_2022_34715_MOESM2_ESM.pdf]

Descriptions of additional Supplementary files

Supplementary Movie 1. **mDia1-nucleated F-actin network growth.** 83 nM mDia1-nucleated F-actin network growth on the lipid bilayer. Scale bar is 10 μm.

Supplementary Movie 2. **Arp2/3-nucleated F-actin network growth.** 7.4nM Arp2/3-nucleated F-actin network on the lipid bilayer. Scale bar is 25 μm.

Supplementary Movie 3. **mDia1-nucleated F-actin network contraction actin channel.** 83 nM mDia1-nucleated F-actin network contraction actin channel. Scale bar is 10 μm.

Supplementary Movie 4. **mDia1-nucleated F-actin network contraction myosin channel.** 83 nM mDia1-nucleated F-actin network contraction myosin channel. Scale bar is 10 μm.

Supplementary Movie 5. **Photoactivation of Myosin II thick filaments in mDia1-nucleated F-actin network.** 83 nM mDia1-nucleated F-actin and Skeletal Muscle Myosin II thick filaments with Blebbistatin contract when exposed under 405nm laser. Scale bar is 10 μm.

Supplementary Movie 6. **Photoactivation of Myosin II thick filaments in Arp 2/3-nucleated F-actin network.** 74nM Arp 2/3-nucleated F-actin and Skeletal Muscle Myosin II thick filaments with Blebbistatin do not contract when exposed under 405nm laser. Scale bar is 10 μm.

Supplementary Movie 7. **HMM on mDia1-nucleated F-actin network.** Fluorescently labeled HMM (Myosin II) in 83 nM mDia1-nucleated F-actin. Scale bar is 10 μm.

Supplementary Movie 8. **HMM on Arp 2/3-nucleated F-actin network.** Fluorescently labeled HMM (Myosin II) in 0.74nM Arp 2/3-nucleated F-actin. Scale bar is 10 μm.

Supplementary Movie 9. **Laser Ablation on mDia1-nucleated F-actin network actin channel.** 83 nM mDia1-nucleated F-actin aster ablation and retraction. Scale bar is 10 μm.

Supplementary Movie 10. **Laser Ablation on mDia1-nucleated F-actin network myosin channel.** Myosin channel of 83 nM mDia1-nucleated F-actin aster ablation and retraction. Scale bar is 10 μm.

Supplementary Movie 11. **Actomyosin overlay to demonstrate force transmission.** 83 nM mDia1-nucleated F-actin network overlay with Myosin thick filaments. The Myosin thick filaments transmit force through actin filaments. Scale bar is 10 μm.

Supplementary Movie 12. **Myosin motion for Arp 2/3 and mDia1-nucleated F-actin networks.** The motion of Myosin thick filaments on 74 nM Arp 2/3 (left) and 830 nM mDia1-nucleated F-actin. Scale bar is 10 μm.
